# Inflammatory signatures in the spectrum of myeloid diseases

**DOI:** 10.1002/hem3.70428

**Published:** 2026-07-07

**Authors:** Alice Ibbotson, Simon Crouch, Jacqueline Ferrari, Thomas Young, Ann W. Morgan, Anna Gallì, Sara Pozzi, Martina Sarchi, Virginia Camilotto, Martina Boldini, Chiara Elena, Luca Malcovati, Sinisa Savic

**Affiliations:** ^1^ Leeds Institute of Rheumatic and Musculoskeletal Medicine University of Leeds Leeds United Kingdom; ^2^ Epidemiology and Cancer Statistics Group University of York York United Kingdom; ^3^ Fondazione IRCCS Policlinico S. Matteo & University of Pavia Pavia Italy; ^4^ Leeds Institute of Cardiovascular and Metabolic Medicine University of Leeds Leeds United Kingdom; ^5^ NIHR Leeds Biomedical Research Centre Leeds United Kingdom

## Abstract

Dysregulated innate immunity contributes to clonal cytopenias and myeloid neoplasms, but its extent across disease stages and clinical relevance remain incompletely defined. We analyzed plasma ASC/NLRP3 double‐positive (DP) specks, ASC single‐positive (SP) specks, and 45 cytokines in 223 patients with idiopathic cytopenias of undetermined significance (ICUS)/clonal cytopenias of undetermined significance (CCUS), myelodysplastic syndromes (MDS), and chronic myelomonocytic leukemia (CMML) and 39 matched non‐inflammatory controls using adjusted regression, survival modeling, and paired longitudinal analyses. Inflammasome activation and cytokine perturbations were evident across the disease spectrum. DP‐ASC specks were elevated in MDS and CMML, whereas SP‐ASC specks were increased across all groups, indicating activation of ASC‐containing inflammasomes beyond NLRP3. Cytokines followed a graded ICUS → MDS → CMML pattern, with widespread upregulation of interleukins and chemokines (including IL‐7, IL‐8, IL‐11/CXCL11, and CCL7) alongside suppression of stem and progenitor support factors such as CSF3, FLT3LG, TRAIL, and TWEAK. At baseline, elevated IL‐15 and MMP1 predicted progression to acute myeloid Leukaemia, while higher IL‐10, CXCL8, and IL‐18 were associated with reduced survival; ASC specks were not independently prognostic. Longitudinal increases in selected cytokines distinguished progressors (area under the curve 0.82; 95% CI: 0.49–1.0). Cytokine patterns correlated with mutation categories, with the isolated SF3B1 mutation associated with higher DP‐ASC specks. These findings define early and progressive inflammasome engagement and nominate dynamic cytokine panels and the inflammasome–IL‐1 axis as actionable biomarkers and therapeutic targets.

## INTRODUCTION

Age‐associated clonal expansions of hematopoietic stem and progenitor cells (HSPCs) are increasingly recognized as early drivers along a biological continuum that spans idiopathic cytopenias of undetermined significance (ICUS) and clonal cytopenias (CCUS), through myelodysplastic syndromes (MDS) and chronic myelomonocytic leukemia (CMML).^1^, ^2^, ^3^, ^4^ Although genetic lesions confer a substrate for clonal advantage, accumulating evidence shows that dysregulated innate immunity and chronic inflammation shape clonal fitness, marrow failure, and leukemic evolution.^5^, ^6^ Patients with autoimmune or autoinflammatory disease have a disproportionately high incidence of myeloid neoplasms, supporting the concept that inflammatory signaling contributes directly to disease biology rather than representing a secondary phenomenon.^7^, ^8^, ^9^, ^10^

Innate immune pathways appear central to this process. Toll‐like receptor (TLR) signaling is overexpressed in MDS progenitors, driving NF‐κB activation, aberrant myelopoiesis, and pyroptotic cell death.^11^, ^12^ Alarmins released from pyroptotic precursors amplify sterile inflammation, establishing a self‐sustaining inflammatory loop.^13^ Among canonical inflammasome sensors, the NLRP3 inflammasome has been most strongly implicated in MDS, where ASC recruitment and caspase‐1 activation promote pyroptosis and IL‐1β/IL‐18 secretion.^14^ These cytokines support mutant HSPC survival and expansion in preclinical models.^15^ However, ASC also participates in non‐NLRP3 inflammasomes,^16^ raising the possibility of broader inflammasome involvement across the myeloid spectrum.

Our group recently demonstrated enhanced activation of the NLRP3 inflammasome in lower risk MDS using a novel flow‐based assay that specifically detects ASC/NLRP3 protein specks and showed that activation levels in MDS approximate those seen in classical autoinflammatory diseases.^17^ We also observed distinct profiles of ASC‐only specks, suggesting activation of other ASC‐containing inflammasome complexes. These findings provide direct evidence that clinically measurable inflammasome activation is present in lower risk MDS and may be biologically relevant.

Beyond inflammasome activation, dysregulated cytokine signaling, including IL‐1β, IL‐6, IL‐10, CXCL8, TNF, and interferon‐regulated mediators, has been consistently reported in CCUS, MDS, and CMML, with several mediators associated with clinical outcomes.^18^, ^19^, ^20^ Nevertheless, the diagnostic and prognostic significance of these pathways remains incompletely defined, particularly in early disease.

To address these gaps, we quantified ASC/NLRP3 protein specks (specific to NLRP3 activation), ASC‐only specks, and a panel of 45 cytokines across healthy controls (HC), ICUS/CCUS, MDS, and CMML. Using regression models adjusted for age and sex and incorporating long‐term clinical outcomes, we asked whether (i) inflammatory signatures distinguish disease stages, (ii) inflammasome activation extends across the myeloid spectrum, and (iii) specific cytokines predict progression, transfusion requirement, or survival. Our results demonstrate broad inflammasome activation and cytokine dysregulation across ICUS/CCUS, MDS, and CMML, with discrete biomarkers showing clinical relevance.

## METHODS

### Patient cohort

We studied a prospective cohort of 223 patients diagnosed with CCUS, ICUS, MDS, MDS/MPN, or CMML recruited from the Fondazione IRCCS Policlinico San Matteo and University of Pavia reference centers. Clinical data and blood samples were collected following informed consent from all participants. Diagnostic criteria were updated in accordance with the 2016 and 2022 revisions of the classification of hematopoietic tumors.^21^, ^22^, ^23^ Demographic and clinical details of the patient population are summarized in Table 1. In addition to the core dataset, the following inflammatory and chronic comorbid conditions were recorded: diabetes, inflammatory bowel disease, rheumatic disease, chronic pulmonary disease, atherosclerotic disease, liver disease, nephropathy, obesity, infectious disease, and other autoimmune disorders. These variables were abstracted from medical records as defined and documented by treating specialist teams.

**Table 1:** Summary of patient demographic features and diagnosis.

Disease group	Total	Male	Female	Age	Inflammatory complications	Disease progression
ICUS/CCUS	16% (35)	63% (22)	37% (13)	60 (21–83)	23% (8)	29% (10)
MDS	62% (139)	53% (73)	47% (66)	64 (21–84)	32% (45)	27% (38)
CMML	22% (49)	78% (38)	22% (11)	68 (31–84)	29% (14)	24% (12)
Healthy controls	39	31% (12)	69% (27)	70 (47–93)		

Abbreviations: CCUS, clonal cytopenias of undetermined significance; CMML, chronic myelomonocytic leukemia; ICUS, idiopathic cytopenias of undetermined significance; MDS, myelodysplastic syndromes.

Baseline samples were collected at the time of diagnosis or first referral, while sequential samples were obtained during clinical follow‐up in either a stable (*n* = 163) or progressive disease phase (*n* = 60), with a median interval between samples of 16 months. Progression status was defined relative to the timing of sample collection. Patients were classified as progressors only if disease progression occurred prior to, or at the time of, the follow‑up sample. Progression occurring after the second sample was not considered for this analysis, and such patients were classified as stable at follow‑up. Genomic DNA was extracted from peripheral blood granulocytes using standard protocols for human tissues. All patients underwent a comprehensive hematologic work‐up in accordance with current standards and guidelines. Targeted capture DNA sequencing of recurrently mutated genes in myeloid neoplasms was performed locally using four validated panels. Further details on DNA sequencing and clonal hierarchy analysis are provided in the Supporting Information S2: Supplemental Material.

This study was approved by local ethics committees at Fondazione IRCCS Policlinico San Matteo, Pavia, Italy, and the Leeds Institute for Rheumatic and Musculoskeletal Medicine, Leeds, UK. All the procedures followed were in accordance with the Helsinki Declaration of 1975, regularly revised up to the last 2024 version.

In total, 39 age and sex matched non‐inflammatory HC were recruited from University of Leeds staff and a cataract pre‐assessment clinic (Leeds East Research Ethics Committee 04/Q1206/107). Demographic data of HC are shown in Table 1.

### ASC/NLRP3 specks detection

Using an in‐house flow cytometry assay, we quantified ASC and NLRP3 proteins in patient plasma. Detailed information on assay development has been previously published.^24^ Briefly, 60 μL of plasma was incubated with a R‐phycoerythrin‐conjugated anti‐ASC antibody (BioLegend, 653904; 1:50 dilution) and an APC‐conjugated anti‐NLRP3 antibody (BioTechne, IC7578A; 1:10 dilution) on a plate shaker for 1 h at room temperature. Samples were analyzed using a Beckman Coulter CytoFLEX‐S flow cytometer, with the event rate set to “High”; 30 μL of plasma per sample was acquired. Gating was performed on events approximately 1 μm in size using fluorescently tagged submicron particle size reference beads (Invitrogen, F13839). Double‐positive (DP) events were interpreted as ASC‐NLRP3 inflammasome specks and are referred to throughout the manuscript as DP‐ASC specks. Single‐positive (SP) ASC events are referred to as SP‐ASC specks. Data were processed in Microsoft Excel to subtract background fluorescence and calculate ASC/NLRP3‐positive events/μL and ASC‐only‐positive events/μL.

### Cytokine measurement

Cytokines were measured using the Olink® Target 48 Cytokine panel (Olink Proteomics AB, Uppsala, Sweden) following the manufacturer's instructions by Allotype Biodiscovery based at the University of Leeds. In total, 45 cytokines were quantified from 1 μL of plasma using Proximity Extension Assay (PEA) technology, which has been well described.^25^ The data were quality controlled and normalized to adjust for intra‐ and inter‐run variation using an internal extension control and calibrators. For absolute quantification, the final output is presented as pg/mL using a 4‐Pl. All assay validation data is available on the manufacturer's website (www.olink.com).

For paired analysis, patient data were matched by samples taken initially and samples taken in a follow‐up appointment. Data normality was calculated using the Shapiro–Wilk test; in this case, the distribution of cytokines in all groups was found to be non‐parametric. Significance between cytokine expression in different patient groups was determined using the Wilcoxon signed rank test and corrected using Benjamini–Hochberg, *Q* values < 0.05 were determined to be significant. Statistical analysis was carried out in Python using the scipy stats library.

### Statistical analysis

Analyses included 223 patients with lower risk MDS and 39 controls. Continuous variables were log‐transformed to reduce skewness; group comparisons used linear regression on log‐transformed data, presented as ratios of geometric means with 95% CIs. Models were adjusted for age and sex when appropriate. Cytokine analyses employed both unequal‐variance *t* tests and Mann–Whitney tests, with Bartlett's test assessing variance equality. False discovery rate (FDR) correction was applied using the Benjamini–Hochberg method. Robust (HC3) variance estimators were used to account for heteroscedasticity. Subgroup comparisons (ICUS, CMML, and MDS) were performed relative to controls. Pairwise comparisons presented in Supporting Information S4: Table 2 are exploratory and interpreted in the context of overall group‐level heterogeneity assessed in the primary analysis. Associations between biomarkers and inflammatory comorbidities were examined by logistic regression. Time‐to‐event outcomes (progression to higher risk MDS, acute myeloid Leukaemia (AML) transformation, and overall survival) were analyzed by Kaplan–Meier and Cox proportional hazards models, reporting hazard ratios (HRs) with 95% CIs. Receiver operating characteristic (ROC)–area under the curve (AUC) values were calculated via logistic regression using a 70% training and 30% test set split. Reported values were obtained using only the test set. Analyses were conducted in R (R Foundation for Statistical Computing, Vienna, Austria); P < 0.05 was considered significant.

## RESULTS

### Patient characteristics

A total of 223 patients were included in the study. Of these, 35 were diagnosed with ICUS/CCUS, 139 with MDS, and 49 with CMML. Within the MDS cohort, there were 10 different subtypes. Patient data were compared to an age/sex‐matched cohort of 39 non‐inflammatory HC. The distribution of sex across disease categories, disease‐related complications, and progression status is summarized in Table 1. A more detailed description of the cohort, including genetic and karyotypic data, is provided in Supporting Information S3: Table 1.

### Levels of ASC/NLRP3 specks are significantly elevated across myeloid disease spectrum

We have previously demonstrated that ASC/NLRP3 protein or DP‐ASC specks are significantly elevated in the serum of patients with lower risk MDS compared to HC.^17^ In this study, we sought to determine whether NLRP3 inflammasome activation is also evident across different stages of myeloid malignancy. To this end, we analyzed three patient cohorts representing a spectrum of myeloid disorders: ICUS/CCUS, MDS, and CMML.

**Figure 1:**
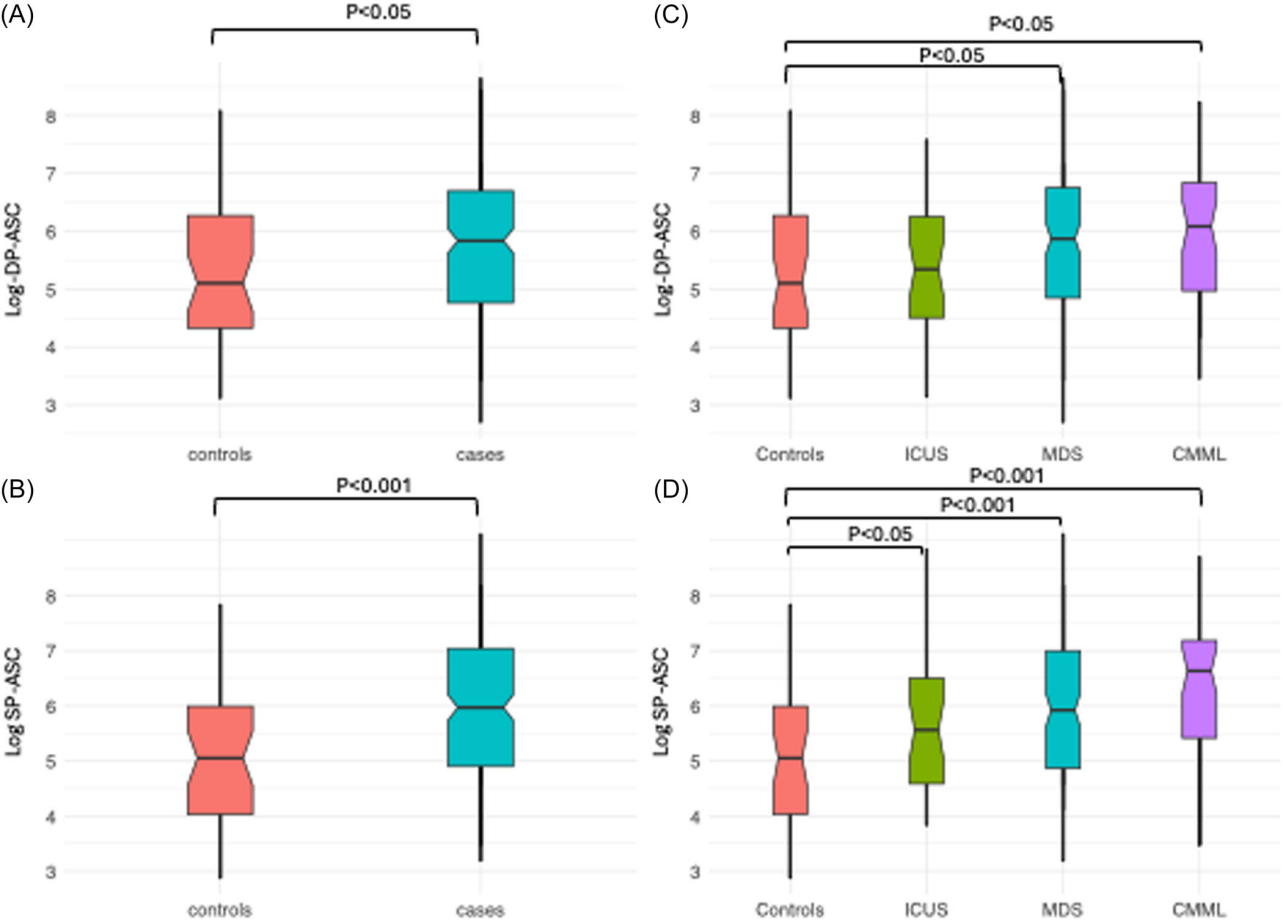
**Single‐positive (SP)‐ASC and double‐positive (DP)‐ASC and specks in lower risk myeloid neoplasms adjusted for age and sex.** Speck formation was quantified in peripheral blood samples from patients with idiopathic cytopenias of undetermined significance (ICUS), myelodysplastic syndromes (MDS), and chronic myelomonocytic leukemia (CMML), and from healthy controls. Both DP‐ASC and SP‐ASC speck values were log‐transformed and compared between diagnostic groups using linear regression models adjusted for age and sex. **(A, B)** DP‐ASC and SP‐ASC specks compared to all cases. **(C, D)** ASC/NLRP3 and ASC specks, respectively, stratified by diagnostic category. Relative to controls, mean ASC/NLRP3 speck levels were increased in CMML (ratio = 1.81, 95% CI 1.15–3.27, P = 0.034) and in MDS (ratio = 1.62, 95% CI 1.06–2.57, P = 0.039). Mean ASC specks were similarly elevated in ICUS (ratio = 2.07, 95% CI 1.12–3.84, P = 0.021), CMML (ratio = 3.42, 95% CI 1.15–3.27, P = 3.1 × 10^−5^), and MDS (ratio = 2.63, 95% CI 1.06–4.57, P = 8.0 × 10^−6^) compared with controls. Age and sex were not significant covariates. Boxes represent interquartile ranges, horizontal lines the medians, and whiskers the 1.5 × interquartile range (IQR) range.

When compared to HC, and when adjusted for age and sex, both DP‐ASC and SP‐ASC specks were significantly elevated across the combined disease groups (P = 0.04 and P = 3.14 × 10^−5^, respectively; Figure 1A,B). Further subgroup analysis revealed that DP‐ASC speck levels were significantly higher in MDS (P = 0.039) and CMML compared to HC, but not in ICUS/CCUS (Figure 1C), with the most pronounced difference observed in CMML (P = 0.034).

Interestingly, a distinct pattern emerged when SP‐ASC specks were analyzed. As in our previous work, SP‐ASC speck levels showed a broader distribution compared to DP‐ASC specks. In contrast to DP‐ASC findings, SP‐ASC specks were significantly elevated across all disease subgroups relative to HC, with greater statistical significance: ICUS/CCUS (P = 0.021), MDS (P = 8.0 × 10^−6^), and CMML (P = 3.1 × 10^−5^) (Figure 1D).

Together, these findings confirm and extend previous studies demonstrating inflammasome activation in myeloid disorders. Moreover, the differential distribution of SP‐ASC versus DP‐ASC specks suggests that ASC inflammasomes other than NLRP3 may contribute to disease pathogenesis.

### A range of cytokines are significantly elevated across a spectrum of myeloid disorders

To further investigate the extent of inflammatory pathway dysregulation in myeloid disorders, we measured plasma levels of 45 cytokines using the Olink® Target 48 Cytokine panel. After adjusting for age, sex, and multiple comparisons, we identified several significant differences between patients and HC. In total, 18 of the 45 cytokines were found to be significantly altered when comparing all cases to HC (Table 2). Highly elevated cytokines in order of significance included EGF, MMP1, CXCL11, CCL7, CXCL8 (IL‐8), IL‐7, CCL13, CCL3, CCL8, and IL1β. Moderately increased cytokines included ORL1, IL‐18, and VEGFA, whilst major decreases were seen in CSF3 (G‐CSF), FLT3LG, TNFSF10 (TRAIL), TNFSF12 (TWEAK), and MMP12. This pattern of cytokine dysregulation suggests heightened myeloid inflammatory activation and immune cell recruitment, coupled with bone marrow microenvironment remodeling, disrupted stem/progenitor support signals, and impaired apoptotic and stress‐response mechanisms.

**Table 2:** Cytokine ratios relative to the healthy control group.

Cytokine	Ratio (95% confidence interval)	P value	Adjusted P value
CCL8	1.7 (1.4, 2.0)	<10^−4^	<10^−4^
IL33	1.6 (0.94, 2.6)	0.088	0.15
CXCL12	1.3 (1.0, 1.7)	0.025	0.059
OLR1	1.5 (1.2, 1.9)	2.1 × 10^−4^	6.7 × 10^−4^
IL27	1.5 (1.0, 2.2)	0.038	0.081
IL2	1 (0.63, 1.7)	0.93	0.93
CXCL9	0.95 (0.7, 1.3)	0.76	0.83
TGFA	1.2 (0.99, 1.4)	0.059	0.11
IL1B	1.7 (1.3, 2.3)	3.5 × 10^−4^	0.0011
IL6	1.1 (0.72, 1.6)	0.76	0.83
IL4	0.52 (0.22, 1.2)	0.12	0.19
TNFSF12	0.8 (0.72, 0.89)	<10^−4^	1.0 × 10^−4^
TSLP	0.86 (0.52, 1.4)	0.54	0.66
CCL11	0.88 (0.74, 1.0)	0.15	0.23
HGF	1.1 (0.93, 1.3)	0.29	0.40
FLT3LG	0.6 (0.47, 0.76)	<10^−4^	1.0 × 10^−4^
IL17F	1 (0.65, 1.7)	0.86	0.88
IL7	2.1 (1.4, 3.0)	1.7 × 10^−4^	5.8 × 10^−4^
IL13	0.73 (0.29, 1.8)	0.49	0.63
IL18	1.4 (1.2, 1.8)	4.1 × 10^−4^	0.0012
CCL13	1.9 (1.6, 2.4)	<10^−4^	<10^−4^
TNFSF10	0.67 (0.58, 0.77)	<10^−4^	<10^−4^
CXCL10	0.84 (0.50, 1.4)	0.51	0.64
IFNG	0.94 (0.68, 1.3)	0.72	0.83
IL10	1.3 (0.96, 1.8)	0.087	0.15
CCL19	0.78 (0.57, 1.1)	0.14	0.22
TNF	1.2 (0.98, 1.4)	0.078	0.15
IL15	1.1 (1.0, 1.2)	0.054	0.11
CCL3	1.8 (1.5, 2.1)	<10^−4^	<10^−4^
CXCL8	2.1 (1.8, 2.6)	<10^−4^	<10^−4^
MMP12	0.74 (0.59, 0.92)	0.0082	0.021
CSF2	1.1 (0.75, 1.6)	0.62	0.74
CSF3	0.52 (0.39, 0.70)	<10^−4^	<10^−4^
VEGFA	1.3 (1.1, 1.5)	0.0085	0.021
IL17C	0.86 (0.58, 1.3)	0.47	0.62
EGF	8.7 (5.2, 14)	<10^−4^	<10^−4^
CCL2	0.93 (0.83, 1.1)	0.27	0.39
IL17A	0.68 (0.32, 1.4)	0.32	0.43
OSM	0.75 (0.54, 1.0)	0.083	0.15
CSF1	1 (0.92, 1.1)	0.85	0.88
CCL4	1.2 (1.0, 1.5)	0.028	0.063
CXCL11	3.1 (2.3, 4.3)	<10^−4^	<10^−4^
LTA	0.98 (0.84, 1.2)	0.85	0.88
CCL7	2.8 (2.3, 3.5)	<10^−4^	<10^−4^
MMP1	4.2 (3.1, 5.7)	<10^−4^	<10^−4^

*Note*: Ratios (95% CI) represent fold differences between cases and controls. Unadjusted comparisons used Welch *t* tests and Mann–Whitney tests, with Bartlett tests guiding test choice; CIs assume the *t* test. Adjusted analyses (age and sex) used models with robust (HC3) standard errors. Benjamini–Hochberg correction was applied for multiple testing.

**Figure 2:**
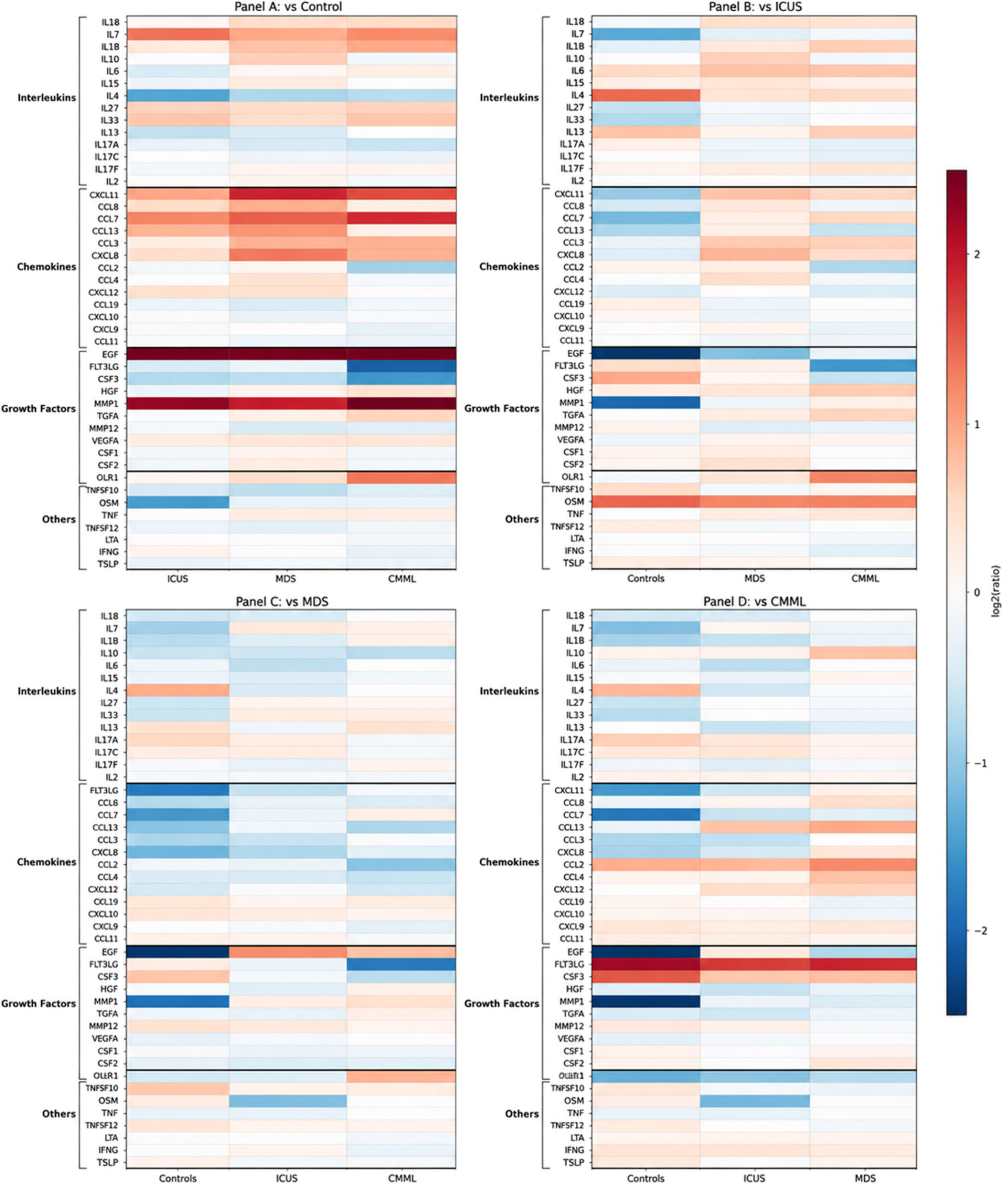
**Cytokine profiles across diagnostic subgroups of lower risk myelodysplastic syndromes (MDS).** Heatmaps display log_2‐_transformed biomarker values (cytokine ratios) summarized as fold change ratios relative to the reference group indicated in each panel. Each panel shows log_2_‐transformed ratios adjusted for age and sex, comparing idiopathic cytopenias of undetermined significance (ICUS), MDS, and chronic myelomonocytic leukemia (CMML) against different reference groups (control, ICUS, MDS, and CMML). Rows represent cytokines grouped by functional class (interleukins, chemokines, growth factors, and others). Color scale indicates magnitude and direction of change (red = upregulated, blue = downregulated). Ratios >1 indicate increased levels, and ratios <1 indicate decreased levels compared with the reference group. These subgroup comparisons are presented as descriptive effect‐size summaries; no multiple‐testing correction was applied, and statistical significance is not inferred.

To determine whether the differences in cytokine levels were uniformly distributed across all myeloid conditions, we conducted a more detailed analysis by comparing cytokine levels in each myeloid subgroup (ICUS/CCUS, MDS, and CMML) separately with those in HC. Simultaneous modeling of ICUS, MDS, and CMML demonstrated overall heterogeneity in cytokine profiles across diagnoses, supporting subsequent diagnosis‐specific contrasts. Across the three disease states, a broad pattern of cytokine dysregulation was observed, with several markers showing progressive alteration from ICUS to MDS and CMML. Strong and consistent relative elevations were seen in inflammatory chemokines, including CCL7, CCL8, CXCL11, CCL3, and CXCL8, which had higher relative levels in nearly all groups. Key pro‐inflammatory mediators such as IL‐1β and IL‐18 were also showed a trend towards elevation in MDS and CMML, reflecting heightened myeloid activation. Markers associated with stromal and extracellular matrix remodeling, particularly MMP1, showed large fold increases across all disease states, whereas MMP12 was reduced in MDS and CMML, and angiogenic factors such as VEGFA and HGF were increased in the more advanced conditions. In contrast, cytokines critical for hematopoietic stem and progenitor support, including CSF3 and FLT3LG, were consistently and relatively decreased across all groups, with the most profound suppression seen in CMML. Apoptosis‐ and stress‐response mediators TNFSF10 (TRAIL) and TNFSF12 (TWEAK) were likewise reduced in all disease states. Notably, EGF exhibited the strongest relative increase overall, particularly in ICUS and CMML. Together, these findings indicate a shared but progressively intensifying cytokine signature across ICUS, MDS, and CMML, characterized by amplified inflammatory and chemotactic signaling, remodeling of the bone marrow microenvironment, and marked disruption of stem/progenitor‐supportive and apoptotic pathways. The overall pattern of the cytokine differences across the disease groups compared to HC is shown in Figure 2A and a detailed breakdown in Supporting Information S4: Table 2. We also conducted further analyses to examine differences between the individual diagnostic groups. These are presented as descriptive, exploratory analyses to illustrate effect‐size patterns, since corrections for FDR were not performed due to the relatively small sample size. When comparing ICUS/CCUS as premalignant stages to the malignant diagnostic groups, a similar pattern was observed. ICUS/CCUS already displays clear cytokine abnormalities, especially suppression of growth‐factor and stromal pathways. MDS shows broad activation of inflammatory cytokines and chemokines, whilst CMML again is associated with the most severe dysregulation, with maximal inflammation, chemokine upregulation, and marked suppression of stem‐cell supportive signals (Figure 2B–D and Supporting Information S5: Table 3, Supporting Information S6: Table 4, and Supporting Information S7: Table 5). This supports a model in which increasing inflammatory disruption and microenvironmental remodeling accompany clonal progression across myeloid disease states.

### The association between genetic drivers, NLRP3 inflammasome activation, and cytokine dysregulation

Myeloid disorders are associated with a wide range of genetic drivers. To explore potential mechanistic links, we investigated whether the observed ASC specks and cytokine dysregulation were associated with specific genetic mutation profiles. We explored the links with the individual mutations and grouped them based on their functional similarities into seven distinct sets. These included: chromatin and histone modifiers, cohesin complex, DNA methylation, RNA splicing, signaling, transcription regulation, and tumor suppression. For a detailed list of the mutations included in each category, please refer to the Methods section.

We first tested for associations between genetic mutations and ASC/NLRP3 speck formation. To reduce the risk of overfitting, the analysis was limited to genes with mutations identified in at least 10 individuals. In this subset, no significant associations were observed, regardless of whether the data were adjusted for age and sex, or if we analyzed DP‐ASC or SP‐ASC specks separately. Similarly, we did not find any significant associations with any of the gene sets.

Interestingly, when the analysis was restricted to patients who had *SF3B1* mutation in isolation, after adjusting for age and sex, we found a significant association with DP‐ASC specks (P = 0.0062).

**Figure 3:**
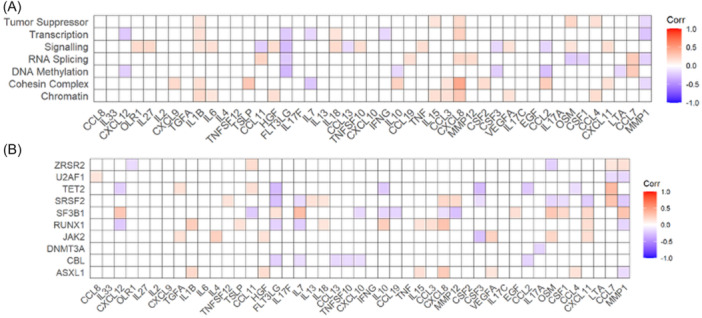
**Cytokine–mutation correlation matrices. (A)** Pearson correlation heatmap between cytokine levels and mutation pathway categories. Red indicates positive correlation; blue indicates negative correlation. Signaling and chromatin pathways show broad positive correlations with pro‐inflammatory cytokines (e.g., IL‐1β, IL‐6, CXCL8, and CCL4), whereas DNA methylation and RNA splicing pathways display negative correlations with stem/progenitor‐supportive cytokines (FLT3LG, CSF3, and CXCL12). **(B)** Correlation heatmap for individual driver genes. ASXL1, RUNX1, SRSF2, and JAK2 mutations associate with increased inflammatory cytokines, while TET2 shows marked suppression of FLT3LG, CSF3, and CXCL12. Spliceosome mutations exhibit distinct cytokine signatures. Correlation coefficients range from −1 to +1.

Analysis of cytokine–mutation associations revealed that distinct mutational pathways were linked to highly divergent inflammatory profiles. Chromatin and cohesin gene mutations showed the strongest positive correlations with pro‐inflammatory cytokines, including IL‐1β, IL‐6, CXCL8, CCL3, CCL4, and HGF, indicating robust activation of innate immune and chemokine‐driven recruitment programs. In contrast, mutations affecting DNA methylation and transcriptional regulators were predominantly associated with reduced levels of key niche‐supportive cytokines, most notably CXCL12, FLT3LG, and CSF3, consistent with impaired progenitor maintenance and attenuated stromal signaling. Interestingly, mutations in transcription regulation genes also correlated with increased levels of pro‐inflammatory cytokines, IL‐1β, IL‐18, and CXCL8. Splicing factor mutations demonstrated a mixed pattern, combining suppression of progenitor‐support cytokines (FLT3LG, CSF1) with increases in chemokines associated with leukocyte trafficking (CCL7, CCL11) and extracellular matrix remodeling (MMP12). Signaling pathway mutations correlated with broad upregulation of inflammatory mediators, including IL‐1β, IL‐6, IL‐18, TNF, and VEGFA, reflecting heightened inflammasome and stromal activation. Tumor‐suppressor mutations were associated with a more moderate but consistent inflammatory skew, marked by increases in IL‐1β, CXCL8, IL‐15, and OSM. Together, these findings demonstrate that cytokine dysregulation in myeloid disorders is not uniform but instead might be associated with mutation‐specific immune and stromal programs, with certain gene sets driving intense inflammatory activation while others predominantly disrupt marrow niche support. (Supporting Information S8: Table 6 and Figure 3A).

In addition, analysis of cytokine–mutation relationships revealed distinct and gene‐specific inflammatory signatures across recurrent myeloid driver mutations. Mutations in *ASXL1*, *RUNX1*, *SRSF2*, and *JAK2* showed the strongest positive associations with pro‐inflammatory cytokines, including IL‐1β, IL‐18, CXCL8, CCL3, and angiogenic mediators such as HGF and VEGFA, indicating robust activation of innate immune pathways and myeloid chemokine networks. *RUNX1* and *ASXL1* mutations in particular were associated with marked increases in IL‐1β and CXCL8, while *SRSF2* mutations correlated with elevated CCL7, CXCL8, and MMP12, consistent with enhanced monocyte recruitment and extracellular matrix remodeling. In contrast, mutations in *TET2* were predominantly associated with suppression of key hematopoietic niche cytokines, most notably CXCL12, FLT3LG, and CSF3, highlighting impaired progenitor support and stromal dysfunction. Spliceosome mutations exhibited heterogeneous patterns: *SF3B1* was associated with increased niche factors (CXCL12, IL‐7) but reduced interferon‐related chemokines (CCL11, CXCL10), whereas *U2AF1* and *ZRSR2* were linked to reductions in matrix‐ and stromal‐derived cytokines (MMP1 and OSM, respectively). Overall, these findings demonstrate that individual driver mutations are associated with distinct inflammatory, chemotactic, and stromal signaling programs, with some mutations (*ASXL1*, *RUNX1*, and *SRSF2*) driving strong inflammatory activation, while others (*TET2*) predominantly disrupt marrow niche support (Supporting Information S9: Table 7). The overall pattern of significant cytokine differences is shown in Figure 3B.

Multivariable modeling to further assess associations between genetic mutations and cytokine levels was not performed due to strong potential collinearity between mutation categories and diagnostic groups, small sample sizes within mutation strata, and the resulting high risk of overfitting.

### Association between the baseline levels of ASC specks and cytokines with inflammatory complications and disease outcomes

Given the well‐established association between myeloid disorders and inflammatory conditions, we explored whether levels of ASC specks or specific cytokines were associated with the presence of inflammatory complications. Identifying such associations could highlight candidate biomarkers for future validation studies. We performed logistic regression analyses to examine these relationships across all documented inflammatory complications, with additional focused analyses on specific conditions such as atherosclerosis and inflammatory rheumatological disorders, since these conditions have a well‑established epidemiological and mechanistic association with clonal hematopoiesis. These analyses did not reveal any statistically significant associations, even when models were unadjusted for age and sex.

We then used Cox regression analysis to assess whether baseline levels of ASC/NLRP3 specks or cytokines at baseline could predict clinical outcomes, including disease progression and overall survival. DP‐ASC and SP‐ASC speck levels were not predictive of either outcome. In contrast, several cytokines showed statistically significant associations. Specifically, elevated levels of IL‐15 (P = 0.0017) and MMP1 (P = 0.0017) were associated with progression from MDS to AML. Additionally, three cytokines, IL‐10, CXCL8, and EGF, approached statistical significance, each with P‐values around 0.06 (Supporting Information S10: Table 8 and Supporting Information S11: Table 9). Further analysis was performed to find predictors of survival. Higher levels of IL‐10 HR 1.4 (95% CI: 1.1–1.6) P = 0.02, CXCL8 HR 1.4 (95% CI:1.1–1.7) P = 0.02, and IL‐18 HR 1.9 (95% CI: 1.3–2.8) P = 0.03 were all predictive of poor survival (Supporting Information S12: Table 10). In addition, high levels of IL‐15 and CCL3 reached near significance, P = 0.063 and P = 0.110, respectively. These analyses should be interpreted as exploratory. All models were univariable (adjusted for age and sex only), and no multivariable modeling incorporating established clinical risk factors was performed. Therefore, the findings do not provide evidence that these biomarkers are independent predictors of outcome beyond existing prognostic models and will require validation in larger, appropriately adjusted cohorts.

### Prediction of disease outcomes based on serial cytokine levels

To assess whether dynamic changes in cytokine levels could help predict disease course, we analyzed paired samples collected at baseline (diagnosis) and at follow‐up (in a phase of stable [*n* = 59] vs. progressive [*n* = 55] disease).

**Figure 4:**
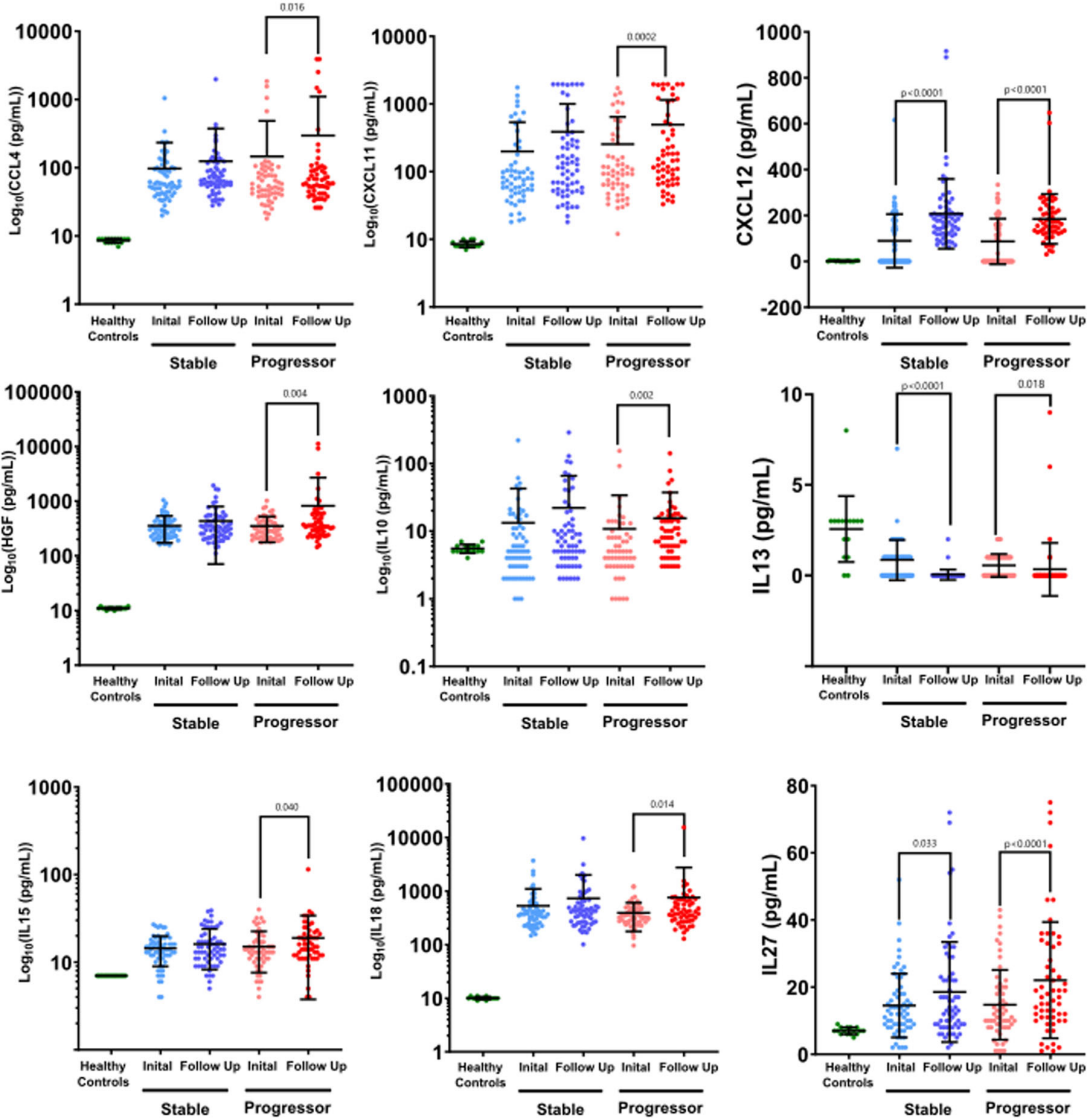
**Initial compared to follow‐up cytokine levels grouped by stable and progression.** Highlighted cytokines showing expression in initial and follow‐up groups, significance determined by false discovery rate (FDR) *Q* < 0.05 calculated using Benjamini–Hochberg (P value determined using Wilcoxon Test [signed rank], data is non‐parametric as determined by Shapiro–Wilk test).

**Figure 5:**
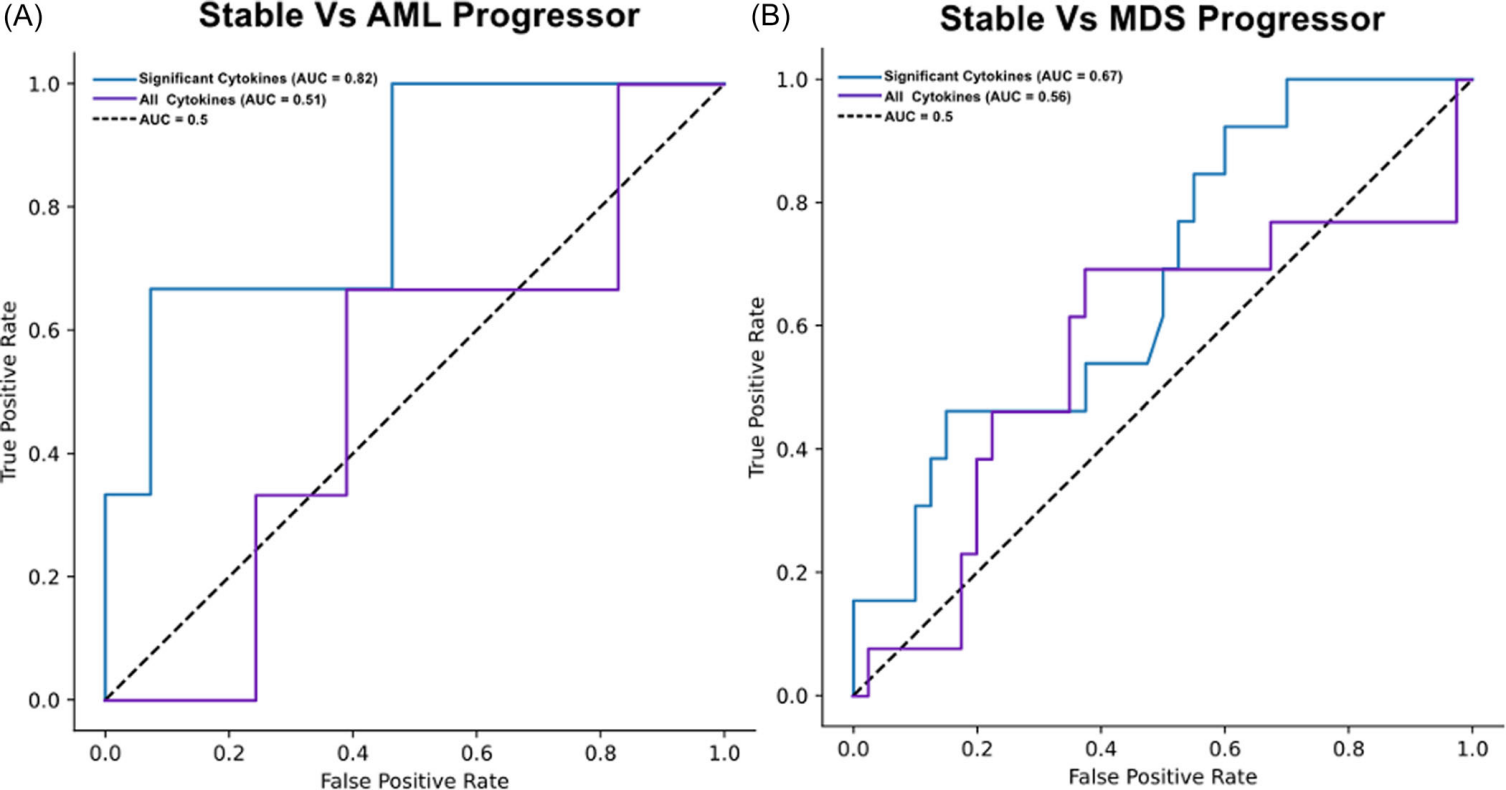
**Receiver operating characteristic (ROC) analysis of cytokine‐based progression models.** ROC curve, using a logistic regression model trained on a random 30% subset of patients, predicting whether the patient is in the stable or **(A)** myelodysplastic syndromes (MDS) progressor/**(B)** AML progressor group, based on cytokine concentrations from follow‐up samples. The blue curve reflects the model using only cytokines previously identified as upregulated in progressors (CCL4, CXCL11, HGF, IL10, IL15, and IL18), while the purple curve represents the model using all cytokines in the Target 48 panel. The dashed line represents the line of no discrimination (AUC = 0.5). AML, acute myeloid leukemia; AUC, area under the curve.

We first analyzed cytokine expression in follow‐up samples grouped by progression status and found no significant differences between stable patients and those who progressed (Supporting Information S1: Figure 1). This suggested that a cross‐sectional view was insufficient, prompting a longitudinal approach. Paired analysis of initial and follow‐up samples using a Wilcoxon signed‐rank test with Benjamini–Hochberg correction showed significant changes in levels of several cytokines over time, including CCL4, CXCL11, CXCL12, HGF, IL‐10, IL‐13, IL‐15, IL‐18, and IL‐27. Notably, when stratifying by progression status, CCL4, CXCL11, HGF, IL‐10, IL‐15, and IL‐18 increased only in patients who progressed, whereas no cytokine changes were unique to stable patients (Figure 4). Whether these changes are causative or symptomatic remains unclear, but they may serve as markers of disease evolution. ROC–AUC analysis using follow‐up cytokine expression demonstrated that this panel discriminated stable versus AML progressors with good accuracy (AUC = 0.82; 95% CI: 0.49–1.0) and stable versus MDS progressors with modest accuracy (AUC = 0.67; 95% CI: 0.51–0.84) (Figure 5). These findings highlight a progression‐associated cytokine signature dominated by chemokines and interleukins involved in immune activation and growth‐factor signaling, suggesting potential utility for tracking disease and identifying patients at risk of progression. Further validation in independent cohorts and evaluation of baseline or dynamic changes will be essential to determine predictive value.

## DISCUSSION

Our study shows that abnormal inflammasome activity and cytokine remodeling are already established at the stage of unexplained cytopenias and intensify progressively through MDS to CMML. ASC/NLRP3 double‐positive (DP‐ASC) specks, a readout of NLRP3 activation, were increased in MDS and CMML, while ASC single‐positive (SP‐ASC) specks were elevated across all disease groups, including ICUS/CCUS. In parallel, we identified a graded cytokine signature characterized by broad upregulation of interleukins and chemokines and consistent suppression of stem/progenitor‐supporting factors (CSF3, FLT3LG, TRAIL, and TWEAK), with CMML showing the most profound disruption. Baseline levels of selected cytokines (notably IL‐15 and MMP1 for AML progression; IL‐10, CXCL8, and CCL13 for survival) and dynamic rises in CCL4, CXCL11, HGF, IL‐10, IL‐15, and IL‐18 in progressors further highlight a tight link between inflammatory signaling and clinical evolution.

Our findings extend prior work that placed the NLRP3 inflammasome at the center of MDS biology. Cumulative evidence suggests that S100A9‐driven NLRP3 activation and pyroptosis underlie ineffective hematopoiesis in MDS, creating a self‐amplifying loop of DAMP release, myeloid‐derived suppressor cell (MDSC) expansion, and chronic inflammatory stress.^13^ Ward et al. subsequently showed that oxidized mitochondrial DNA, released from pyroptotic cells, engages TLR9 and MyD88 to amplify NLRP3 activation in MDS HSPCs, reinforcing this feed‐forward circuit and further impairing hematopoietic function.^26^

Our data agree with this model and broaden it in clinically relevant ways. First, by using ASC/NLRP3 speck detection in plasma, we demonstrate that measurable inflammasome activation is not restricted to overt MDS but is already increased in patients with ICUS/CCUS and rises further in CMML, mirroring disease stage and myeloid burden. Second, the stronger and more pervasive elevation of SP‐ASC specks across all diagnostic groups could indicate that other ASC‐containing inflammasomes (e.g., AIM2, NLRP1, and NLRC4) might be engaged alongside NLRP3. This aligns with mechanistic work showing that ASC is a shared adapter for multiple inflammasome platforms and that extracellular ASC specks can themselves propagate inflammatory signaling.^27^, ^28^ However, as we cannot at this stage exclude the possibility that the observed differences between SP‑ and DP‑ASC speck levels are influenced by assay sensitivity, or by processes such as non‑canonical inflammasome activation, cellular stress, pyroptosis, or extracellular ASC persistence, SP‑ASC specks are best regarded as a surrogate marker of ASC engagement.

The observation that ASC specks are already abnormal in ICUS/CCUS supports the view that inflammasome activation is an early feature of clonal hematopoiesis rather than a late by‐product of marrow failure. Reviews of innate immune–inflammatory signaling in CH and AML have emphasized that chronic pattern‐recognition receptor (PRR) engagement, coupled with age‐related “inflamm‐aging,” can confer a selective advantage to mutant HSPC clones, promote genomic instability, and bias differentiation towards myelomonocytic lineages.^29^ Our data provide a biochemical correlate of this concept in human premalignant disease. Moreover, they dovetail with ex vivo work demonstrating heightened NLRP3 responsiveness in monocytes from older individuals, and a dichotomous, IL‐1β–high subset of CMML patients in whom exaggerated inflammasome activation associates with more severe disease.^30^

That ASC specks did not independently predict progression or survival in our cohort suggests that inflammasome activation may represent a relatively stable disease trait, shaped by age, clonal architecture, and microenvironment, whereas cytokine outputs are more dynamic and tightly coupled to short‐term clinical behavior. This division of labor is biologically plausible: the inflammasome defines the capacity for IL‐1 family signaling and pyroptosis, but moment‐to‐moment cytokine levels are also influenced by infection, comorbidity, and treatment.

We observed a broad inflammatory and chemotactic signature spanning ICUS/CCUS, MDS, and CMML, with stepwise amplification along this continuum. IL‐7, CXCL8 (IL‐8), CXCL11, CCL7, EGF, and MMP1 were consistently elevated across all groups, whereas CSF3, FLT3LG, TRAIL, and TWEAK were uniformly suppressed. CSF3 and FLT3LG are key regulators of normal HSPC maintenance, proliferation, and differentiation, and their suppression^31^, ^32^ suggests a microenvironment that is increasingly unsupportive of healthy hematopoiesis, potentially favoring the competitive advantage of mutant clones. In parallel, reduced levels of TRAIL and TWEAK, cytokines involved in immune‐mediated apoptosis, stress responses, and tissue homeostasis,^33^, ^34^ may reflect impaired immune surveillance and diminished elimination of damaged or aberrant progenitor cells. This pattern suggests simultaneous enhancement of myeloid‐biased inflammatory recruitment and remodeling of the bone‐marrow niche, alongside depletion of factors that support normal stem/progenitor survival and apoptotic pruning. CMML displayed the most extreme phenotype, with maximal chemokine induction, highest MMP1, and the deepest suppression of CSF3/FLT3LG, consistent with its clinical presentation as an inflammatory, monocytic neoplasm with high transformation risk.

These observations are concordant with and extend previous cytokine studies in early myeloid disease. Nielsen et al. reported that patients with ICUS and CCUS already display elevated IL‐6, TNF‐α, IL‐10, and CXCL10, and reduced TGF‐β1, CCL5, and S100A4, to a degree comparable to lower risk MDS.^35^ A high aggregate cytokine load predicted worse survival, independent of mutational status. We corroborate the concept that inflammatory remodeling is present before MDS is morphologically apparent, but by using an expanded panel, we demonstrate that this extends well beyond a limited set of classical cytokines to include IL‐7, CXCL8, IL‐18, multiple CC and CXC chemokines, growth factors (EGF, HGF), and matrix‐modifying enzymes.

The prominence of IL‐1 family signaling in our data is notable. IL‐1β and IL‐18 were both elevated; IL‐18 rose dynamically in patients who progressed, and IL‐18 was a predictor of survival. This is consistent with murine work showing that IL‐1β and IL‐18 directly impair the function of marrow mesenchymal stromal cells (MSCs) in an age‐appropriate MDS model, reducing their ability to support HSPCs and erythroid progenitors.^36^ Inhibition of IL‐1R1, IRAK4, or NLRP3 in that system reversed MSC proliferation abnormalities, restored niche function, and selectively suppressed MDS clones while sparing normal hematopoiesis.^36^ Together with our human plasma data, these findings strongly argue that IL‐1–family cytokines link inflammasome activation to microenvironmental damage and clonal fitness in both early and established myeloid disease.

Genetic data also support a causal connection. IL‐1B rs16944 polymorphisms (GG genotype) confer increased MDS risk, are associated with more complex karyotypes and lower hemoglobin levels, implicating germline variation in IL‐1β production in disease susceptibility and severity.^37^ Our observation that IL‐18 is part of a progression‐associated cytokine panel reinforces the notion that genetically and inflammasome‐driven IL‐1–family signaling is woven into the pathogenesis of these disorders.

Baseline cytokine levels carried prognostic information in our cohort: higher IL‐15 and MMP1 were associated with subsequent AML transformation, while elevated IL‐10, CXCL8, and IL‐18 predicted inferior overall survival. These associations fit with broader literature linking CXCL8 and IL‐18 to poor prognosis in other malignancies through recruitment of MDSCs.^38^, ^39^ IL‐15 promotes survival and proliferation of NK and T cells but can also enhance myeloid expansion and resistance to apoptosis^40^, ^41^; MMP1 and related metalloproteinases remodel the extracellular matrix and may facilitate niche changes favoring malignant clones.^42^

The longitudinal analyses are particularly informative. Increases in CCL4, CXCL11, HGF, IL‐10, IL‐15, and IL‐18 were confined to patients who progressed, and a model incorporating these markers achieved good discrimination for AML transformation (AUC 0.82; 95% CI: 0.49–1.0). This suggests that serial cytokine profiling can capture dynamic shifts in the inflammatory milieu that herald acceleration, in a way that static inflammasome readouts cannot. The inclusion of both T‐cell/NK‐cell–linked cytokines (IL‐15), regulatory mediators (IL‐10), chemokines (CCL4, CXCL11), IL‐1–family signaling (IL‐18), and niche‐related growth factors (HGF) underscores that progression is accompanied by coordinated changes across innate, adaptive, and stromal compartments rather than a single‐axis perturbation.

Our findings align conceptually with Nielsen et al., who showed that a high systemic cytokine load across a more limited panel was associated with shortened survival in CCUS and MDS.^35^ We extend this by (i) demonstrating that specific cytokines (rather than aggregate burden alone) carry prognostic information, and (ii) showing that changes in cytokine levels over time sharpen discrimination of progressors from stable patients.

We observed associations between mutational categories and cytokine patterns, with signaling‐pathway mutations showing the broadest impact and *TET2* mutations in particular associated with reduced FLT3LG, CSF3, CXCL12, IL‐10, and CCL2/4, and higher levels of several chemokines and growth factors. These data echo experimental models in which loss of *TET2* in myeloid cells drives hyper‐responsiveness to inflammatory stimuli and overproduction of IL‐1β and TNF, thereby promoting clonal expansion and skewing towards myelomonocytic differentiation.^43^, ^44^

Of particular interest, an isolated *SF3B1* mutation was associated with higher DP‐ASC specks, suggesting a link between this otherwise relatively indolent genotype and inflammasome activation. *SF3B1*‐mutant MDS is recognized as a distinct subtype with ring sideroblasts, ineffective erythropoiesis, and generally favorable prognosis, although progression can occur in the presence of specific cooperating lesions.^45^ Our data raise the possibility that even in this clinically “good‐risk” category, innate immune activation is measurable and may contribute to phenotypic features such as ineffective erythropoiesis; whether this has therapeutic implications (e.g., for combining erythroid‐targeted agents with anti‐inflammatory strategies) merits investigation.

More broadly, our findings support the notion that highly heterogeneous genetic lesions converge on shared inflammatory endpoints, as proposed in recent reviews of CH and myeloid malignancies.^13^, ^29^ Differences in cytokine signatures across mutation classes may help explain why some clones preferentially expand in inflammatory environments and why patients with apparently similar mutational profiles have divergent clinical courses.

Taken together, these data reinforce the concept that inflammasome–IL‐1–cytokine circuits are not epiphenomena but integral components of disease biology from ICUS/CCUS through MDS to CMML. This has therapeutic implications. The IL‐1/NLRP3 axis is increasingly druggable. In the NUP98‐HOXD13 murine MDS model, blockade of IL‐1R1, IRAK4 or NLRP3 corrected MSC dysfunction and selectively curtailed MDS clones.^36^

Further analysis of a large clinical trial that looked at the effects of IL‐1β inhibition on the prevention of cardiovascular events found that the protective effect of IL‐1β inhibition was greatest in patients who harbored a *TET2* mutation and CH. Furthermore, canakinumab treatment resulted in higher hemoglobin increment in patients with concurrent CH mutations and anemia than patients with CH mutations without anemia or without CH mutations.^46^ Our demonstration of widespread IL‐1 family engagement and NLRP3‐linked ASC specks across early myeloid disease strengthens the rationale for testing of IL‐1 and IL‐18 targeted therapies or NLRP3 inhibitors as disease‐modifying agents in ICUS/CCUS and lower risk MDS.

## LIMITATIONS AND FUTURE DIRECTIONS

This study has limitations. It is observational and cannot establish causality between inflammasome activation, cytokine shifts, and disease progression. Treatment heterogeneity, intercurrent infections, and comorbid inflammatory conditions, although recorded and modeled, may still confound some associations. Sample size for certain subgroup and longitudinal analyses, particularly specific mutation combinations and transformation events, remains modest. Furthermore, some of our statistical analyses were limited by the number of events. Therefore and our findings should be interpreted as exploratory and require validation in independent cohorts, to confirm their prognostic relevance. Finally, plasma biomarkers may not fully reflect the complexity of the marrow microenvironment, including cell‐specific production and spatial organization of inflammatory signals.

Despite these caveats, by integrating ASC speck assays, broad cytokine profiling, genomic data, and clinical outcomes across ICUS/CCUS, MDS, and CMML, we provide direct evidence that inflammasome engagement and cytokine remodeling are early, pervasive, and clinically relevant features of the myeloid disease spectrum. These data, together with converging mechanistic and translational studies from murine models and human cohorts, support a paradigm in which targeting innate immune–inflammatory circuits, particularly the NLRP3–IL‐1 axis and selected chemokine/growth‐factor pathways, could complement mutation‐directed therapies to prevent or delay leukemic transformation and improve outcomes in early myeloid disease.

## AUTHOR CONTRIBUTIONS

**Alice Ibbotson**: Investigation; writing—original draft; formal analysis; writing—review and editing; visualization. **Simon Crouch**: Formal analysis; data curation; visualization; writing—review and editing; methodology. **Jacqueline Ferrari**: Writing—review and editing; investigation; formal analysis. **Thomas Young**: Investigation; writing—review and editing; formal analysis; visualization. **Ann W. Morgan**: Writing—review and editing; resources. **Anna Gallì**: Resources; investigation; writing—review and editing. **Sara Pozzi**: Investigation; writing—review and editing. **Martina Sarchi**: Investigation; writing—review and editing. **Virginia Camilotto**: Investigation; writing—review and editing. **Martina Boldini**: Investigation; writing—review and editing. **Chiara Elena**: Investigation; writing—review and editing. **Luca Malcovati**: Investigation; resources; writing—review and editing; project administration; supervision. **Sinisa Savic**: Writing—original draft; writing—review and editing; visualization; project administration; resources; supervision; formal analysis.

## CONFLICT OF INTEREST STATEMENT

S.S. has the following COI to declare. Study PI: Novartis, Pharming, KalVista, Celldex, Astria, and CSL Behring; advisory boards, lectures: Novartis, Pharming, Takeda, and CSL Behring; Phavaris, KalVista, Otsuka, SOBI, GSK, and AstraZeneca; conference travel support: Novartis; and research grants: Takeda, Pharming, Novartis, and CSL Behring. All other authors have no COI to declare in relation to this manuscript.

## DISCLOSURES

During the preparation of this work, the author(s) used Microsoft M365 Copilot in order to produce a summary of key findings, but not data interpretation. The same AI tool was used to improve text readability but not for generating the content. After using this tool/service, the author(s) reviewed and edited the content as needed and take(s) full responsibility for the content of the published article.

## FUNDING

This work was supported by the Associazione Italiana per la Ricerca sul Cancro (31013), European Hematology Association (202412‐07382), Kennedy Trust for Rheumatology Research, Medical Research Council (MR/Y011945/1), and Leeds Biomedical Research Centre (NIHR203331).

## Supporting information

Supplementary Figure 1**. Baseline cytokine differences between healthy controls (HC), stable patients, and progressors.** Violin plots showing baseline plasma concentrations of selected cytokines in HC, patients who remained clinically stable, and patients who subsequently progressed. Cytokines displayed include CXCL8, EGF, IL‐7, CCL13, CCL3, CCL7, MMP1, TNFSF12 (TWEAK), CCL8, CSF3 (G‐CSF), CXCL11, and VEGFA. Progressors exhibited significantly higher levels of multiple pro‐inflammatory cytokines and chemokines (e.g., CXCL8, CCL13, CCL3, MMP1, and VEGFA) and lower levels of stem/progenitor‐supportive factors (e.g., CSF3, TWEAK) compared with both HC and stable patients. Statistical significance was determined using standard FDR *Q* values (<0.05) using the Benjamini–Hochberg correction (P value determined using Kruskal–Wallis with Dunn's comparison, data is non‐parametric as determined by Shapiro–Wilk test).

Supporting Information.

Supporting Information.

Supporting Information.

Supporting Information.

Supporting Information.

Supporting Information.

Supporting Information.

Supporting Information.

Supporting Information.

Supporting Information.

Supporting Information.

## Data Availability

The datasets generated and/or analyzed during the current study are available from the corresponding author on reasonable request. No identifiable or sensitive information is included in the shared materials.
