# Supplementary material for: Inflammatory signatures in the spectrum of myeloid diseases
Source: Hemasphere. 2026 Jul 7;10(7):e70428. doi: 10.1002/hem3.70428 (PMC13340139; doi:10.1002/hem3.70428)
Supplement: Supplementary file 2 — Supporting Information. [file HEM3-10-e70428-s003.docx]

**Supplementary methods**

Statistical analysis

Analyses included 223 patients with lower-risk myelodysplastic syndromes (MDS) and 39 healthy controls. Both ASC/NLRP3 and ASC speck measurements were available for most participants, and cytokine concentrations were obtained for all cases and a subset of controls. Distributions of continuous variables were examined visually and found to be right-skewed; therefore, values were natural log–transformed prior to analysis. Log-transformed data were approximately normally distributed and were used in all parametric tests.

Group comparisons of ASC/NLRP3 and ASC speck values were performed using linear regression on log-transformed data, with and without adjustment for age and sex. Results are presented as ratios of geometric means with 95% confidence intervals (CIs). The potential influence of age and sex was evaluated in all models but was not statistically significant.

For cytokine analyses, ratios of geometric means were estimated between groups using regression models fitted to log-transformed cytokine levels. Because of the small control sample size and differences in distributional form, both unequal-variance t tests and Mann–Whitney (Wilcoxon rank-sum) tests were performed. Equality of variances was assessed using Bartlett’s test; when variances differed, nonparametric results were preferred. To control for multiple comparisons, P values were adjusted using the Benjamini–Hochberg false discovery rate (FDR) procedure. Analyses adjusted for age and sex used robust (HC3) sandwich estimators to calculate CIs and P values, accounting for potential heteroscedasticity.

Comparisons among diagnostic subgroups (ICUS, CMML, and MDS) relative to healthy controls were conducted using regression models on log-transformed data, with and without adjustment for age and sex. Ratios of geometric means with 95% CIs were reported; CIs and p-values were calculated using the robust sandwich estimator (HC3). No multiple testing correction was applied to subgroup analyses. The subgroup comparisons were intentionally presented as descriptive effect‑size summaries rather than inferential hypothesis tests.

For analysis between cytokine levels and mutational profile, Pearson correlation analyses were performed on log‑transformed variables that approximated normal distributions and were used solely for exploratory visualisation, therefore not adjusted for age, sex, disease group, or multiple testing. Adjusted regression models were used for inference.

Associations between speck or cytokine levels and inflammatory comorbidities (rheumatic and atherosclerotic diseases) were examined using logistic regression models adjusted for age and sex.

Time-to-event analyses, including progression to MDS, transformation to AML, and overall survival, were conducted using Kaplan–Meier methods and Cox proportional hazards regression adjusted for age and sex. Hazard ratios (HRs) with 95% CIs were estimated for each biomarker, modelled as continuous log-transformed variables or categorized by distribution tertiles. Model assumptions were verified using Schoenfeld residuals.

All analyses were performed using R (R Foundation for Statistical Computing, Vienna, Austria). Two-sided P values < 0.05 were considered statistically significant.

Myeloid gene sequencing

Somatic mutation analysis was performed by next generation sequencing (NGS) of a core panel of 54 genes selected based on prior implication in myeloid disease, as previously described^1,2,4^. Samples were analyzed using the TruSight Myeloid Sequencing Panel (Illumina, San Diego, CA, USA). The probe set targets 15 full genes (coding exons and splice sites) and 39 hot spot mutation regions across 568 amplicons of 250 bp in length for a 141 kb total genomic content: ASXL1 (12), ATRX (8-10, 17-31), BCOR (all), BCORL1 (all), EZH2 (all), KDM6A (all), KMT2A (5-8), SETBP1 (4), RAD21 (all), SMC1A (2, 11, 16, 17), SMC3 (10, 13, 19, 23, 25, 28), STAG2 (all), DNMT3A (all), IDH1 (4), IDH2 (4), TET2 (3-11), SF3B1 (13-16), SRSF2 (1), U2AF1 (2, 6), ZRSR2 (all), ABL1 (4-6), BRAF (15), CALR (9), CBL (8-9), CBLB (9-10), CBLC (9-10), CSF3R (14-17), FBXW7 (9-11), FLT3 (14-15, 20), GNAS (8-9), HRAS (2-3), JAK2 (12, 14), JAK3(13), KIT(2, 8-11, 13, 17), KRAS (2-3), MPL (10), MYD88 (3-5), NOTCH1 (26-28, 34), NRAS (2-3), PDGFRA (12, 14, 18), PTEN (5, 7), PTPN11 (3, 13), CEBPA (all), CUX1 (all), ETV6 (all), GATA1 (2), GATA2 (2-6), IKZF1 (all), NPM1 (12), PHF6 (all), RUNX1 (all), CDKN2A (all), TP53 (2-11), WT1 (7, 9). The probe pool was hybridized to 250 ng of gDNA upstream and downstream of each region of interest. An extension-ligation reaction extended across the selected region followed by a ligation step. The resulting templates were amplified by PCR and two unique library specific indexes were incorporated. The resulting libraries were normalized to the same concentration using a bead-based procedure enabling pooling of equal amount of libraries. Pooled DNA libraries were loaded onto Illumina MiSeq for cluster generation and 2x250 paired-end sequencing.

Functionally annotated variants were filtered based on the population frequency (>1%) retrieved from public databases (dbSNP, 1000genome, and ESP6500, gnomAD). Synonymous variants and variants located outside protein-coding regions were removed. Variants with low coverage (<30x) or low supporting reads (<10x), and variants with an allelic fraction (VAF) lower than 2%, were filtered. The remaining variants were considered as candidate somatic mutations and were finally tagged as oncogenic using different criteria based on information retrieved from literature, databases of somatic mutations (Cosmic, cbioportal ClinVar), effects on the structure or function, sequence conservation, and *in silico* prediction effect. Variant allele frequency was calculated as the number of variant reads divided by the total reads.

The analysis of NGS data was suitable for Illumina paired-end sequencing data and performed all the typical steps of secondary analysis from reads mapping and refinement^4,5^ to the identification of somatic variant as single nucleotide variants (SNVs) and short insertions/deletions (Indels). For SNVs and Indels, the variant calling step integrated four state-of-the-art somatic variant calling algorithms^6-9^ since such approach allows identifying somatic variants with higher sensitivity and accuracy than using any single algorithm^10,11^

For mutation pathways, the following genes were included in each pathway.

**Chromatin & histones modifier**: ASXL1, ATRX, BCOR, BCORL1, EZH2, KDM6A, KMT2A, SETBP1. **Cohesin complex**: RAD21, STAG2, SMC1A. **DNA methylation**: DNMT3A, IDH1, IDH2, TET2. **RNA splicing**: SF3B1, SRSF2, U2AF1, ZRSR2. **Signalling**: ABL1, BRAF, CALR, CBL, FLT3, GNAS, JAK2, KIT, KRAS, MPL, NRAS, PDGFRA, PTEN, PTPN11. **Transcription regulation**: CEBPA, CUX1, ETV6, GATA1, GATA2, NPM1, PHF6, RUNX1. **Tumour suppressor**: CDKN2A, TP53, WT1.

**References**

1. Papaemmanuil E. et al. Clinical and biological implications of driver mutations in myelodysplastic syndromes. Blood. 2013;122(22):3616–27.

2. Malcovati L. et al. Clinical significance of somatic mutation in unexplained blood cytopenia. Blood. 2017;129(25):3371-3378.

3. Gallì A. et al. Relationship between clone metrics and clinical outcome in clonal cytopenia. Blood. 2021;138(11):965-976.

4. Li H. & Durbin R. Fast and accurate short read alignment with Burrows-Wheeler transform. Bioinformatics. 2009. 25(14):1754–60.

5. McKenna A. et al. The genome analysis toolkit: A MapReduce framework for analyzing next-generation DNA sequencing data. Genome Res. 2010;20(9):1297–303.

6. Cibulskis K. et al. Sensitive detection of somatic point mutations in impure and heterogeneous cancer samples. Nat Biotechnol. 2013;31(3):213–9.

7. Koboldt D.C. et al. VarScan 2: Somatic mutation and copy number alteration discovery in cancer by exome sequencing. Genome Res. 2012;22(3):568–76.

8. Lai Z. et al. VarDict: a novel and versatile variant caller for next-generation sequencing in cancer research. Nucleic Acids Res. 2016;44(11):e108.

9. Fang H. et al. Indel variant analysis of short-read sequencing data with Scalpel. Nat Protoc. 2016;11(12):2529–48.

10. Rashid M. et al. Cake: a bioinformatics pipeline for the integrated analysis of somatic variants in cancer genomes. Bioinformatics 2013;29(17): 2208-2210.

11. Wang, M. et al. SomaticCombiner: improving the performance of somatic variant calling based on evaluation tests and a consensus approach. Scientific reports 2020, 10(12898): 1-16.
