# Supplementary material for: Inflammatory signatures in the spectrum of myeloid diseases
Source: Hemasphere. 2026 Jul 7;10(7):e70428. doi: 10.1002/hem3.70428 (PMC13340139; doi:10.1002/hem3.70428)
Supplement: Supplementary file 3 — Supporting Information. [file HEM3-10-e70428-s005.docx]

Supplementary Table 1

| **Disease Group** | **Total** | **Male** | **Female** | **Age** | **Karyotype** | **Mutated Genes** | **Inflammatory Complications** | **MDS Progression** | **AML Progression** | **Outcome** |
| --- | --- | --- | --- | --- | --- | --- | --- | --- | --- | --- |
| **ICUS** | 34 | 62% (21) | 38% (13) | 60 (21-83) | 45, X, -Y [2] 46, XY, del(7q) | ASXL1, CDKN2A, DNMT3A, IDH1, KRAS, SF3B1, SRSF2, TET2, U2AF1, ZRSR2 | 21% (7) | 29% (10) | 0% (0) | 9% (3) |
| **CCUS** | 1 | 100% (1) | 0% (0) | 69 | N/A | N/A | 100% (1) | 0% (0) | 0% (0) | 0% (0) |
| **MDS del(5q)** | 3 | 33% (1) | 67% (2) | 64 (55-74) | 46, XY, del(5q)  46, XX, del(5q) | SG3B1 | 100% (3) | 0% (0) | 0% (0) | 67% (2) |
| **MDS-SLD** | 5 | 20% (1) | 80% (4) | 71 (64-76) | 46, XX del(12p) 47, XXY | CBL, NOTCH1, SRSF2, TET2, U2AF1 | 60% (3) | 20% (1) | 0% (0) | 0% (0) |
| **MDS-**  **EB1** | 15 | 53% (8) | 47% (7) | 63 (49-78) | 46, XX, del(5q) [2] 46, XY, -7, +der(7)  46, XX, -11, +mar 45, XX, -7 47, XX, +8 | ASXL1, CBL, CUX1, DNMT3A, GATA2, IDH2, JAK2, KRAS, PHF6, PTPN11, RUNX1, SETBP1, SF3B1, SRSF2, TET2, TP53, U2AF1, ZRSR2 | 7% (1) | 33% (5) | 7% (1) | 67% (10) |
| **MDS-**  **EB2** | 21 | 67% (14) | 33% (7) | 64 (27-79) | 47, XY, +8 | ASXL1, BCOR, BCORL1, CEBPA, CSF3R, CUX1, DNMT3A, EZH2, IDH1, IDH2, JAK2, KDM6A, KIT, KRAS, NPM1, NRAS, RUNX1, SF3B1, SRSF2, STAG2, TET2, TP53, U2AF1, ZRSR2 | 19% (4) | 0% (0) | 19% (4) | 62% (13) |
| **MDS-**  **RS-SLD** | 24 | 42% (10) | 58% (14) | 67 (35-84) | 46, XX, t(9;?) 46, XX, del(12p) 46, XX, -3, +mar 46, XX, del(20q) 46, XX, del(12p), rearr.(11q) 46, XX, inv(9) 47, XY, +8 45, X, -Y [2] | CUX1, DNMT3A, IDH2, JAK2, SF3B1, TET2 | 38% (9) | 21% (5) | 4% (1) | 21% (5) |
| **MDS-RS-MLD** | 23 | 74% (17) | 26% (6) | 62 (44-77) | 46, XY, del(2p), del(20q) 45, X, -Y 46, XY, dup(1q), del(7q) 47, XY, +mar | ASXL1, CDKN2A, DNMT3A, GNAS, IDH1, JAK2, SF3B1, SRSF2, TET2, TP53, U2AF1, ZRSR2 | 35% (8) | 39% (9) | 4% (1) | 43% (10) |
| **MDS/MPN-RS-T** | 9 | 44% (4) | 56% (5) | 64 (47-83) | 46, del(12q) 46, XX, del(11q) | ASXL1, ATRX, CBL, IDH1, JAK2, KRAS, MPL, PHF6, SF3B1 | 33% (3) | 0% (0) | 0% (0) | 67% (6) |
| **MDS-MLD** | 37 | 49% (18) | 51% (19) | 60 (21-84) | 46, XX, add(7p) 46, XY, del(22q) 47, XXY, add(2p), del(12q) 46, XX, del(20q) 45, XX, -5, add(7p) 46, XY, del(5q) 47, XX, +8 45, XX, -7 44, X, -21, -Y | ASXL1, BCOR, CBL, CDKN2A, CUX1, DNMT3A, EZH2, NOTCH1, PTPN11, RUNX1, SETBP1, SMC1A, SRSF2, STAG2, TET2, U2AF1, WT1, ZRSR2 | 38% (14) | 27% (10) | 3% (1) | 22% (8) |
| **MDS/MPN-U** | 2 | 50% (1) | 50% (1) | 80 (78-81) | NA | JAK2, SF3B1, TET2 | 50% (1) | 0% (0) | 0% (0) | 0% (0) |
| **MDS-**  **RS-T** | 1 | 0% (0) | 100% (1) | 64 (47-83) | NA | NA | 0% (0) | 0% (0) | 0% (0) | 100% (1) |
| **aCML** | 1 | 100% (1) | 0% (0) | 81 | NA | BCOR, DNMT3A, KRAS, SF3B1, TET2 | 100% (1) | 0% (0) | 0% (0) | 100% (1) |
| **CMML-0** | 38 | 76% (29) | 24% (9) | 68 (31-84) | 46, XX, t(6;9) 45, X, -Y [5] 47, XY, +8 [2] | ASXL1, BRAF, CBL, CDKN2A, CUX1, DNMT3A, ETV6, EZH2, FLT3, IDH1, IDH2, JAK2, KDM6A, KIT, KRAS, KMT2A, NRAS, PHF6, PTPN11, SETBP1, SF3B1, SRSF2, TET2, U2AF1, ZRSR2 | 26% (10) | 26% (10) | 3% (1) | 37% (14) |
| **CMML-1** | 4 | 75% (3) | 25% (1) | 71 (65-83) | 45, X, -Y 47, XX, +8 | ASXL1, CBL, EZH2, NRAS, SRSF2, TET2 | 25% (1) | 0% (0) | 0% (0) | 50% (2) |
| **CMML-2** | 6 | 83% (5) | 17% (1) | 64 (58-72) | 47, XX, +8  47, XY, +8 47, XY, +11 | ASXL1, CUX1, EZH2, IDH1, IDH2, KRAS, MPL, RUNX1, SRSF2, TET27 | 33% (2) | 0% (0) | 17% (1) | 67% (4) |

For progression, only disease state at follow up is counted, not future progression.

In karyotype column, in square brackets is the number of patients with that karyotype.
