# Supplementary material for: Inflammatory signatures in the spectrum of myeloid diseases
Source: Hemasphere. 2026 Jul 7;10(7):e70428. doi: 10.1002/hem3.70428 (PMC13340139; doi:10.1002/hem3.70428)
Supplement: Supplementary file 4 — Supporting Information. [file HEM3-10-e70428-s004.docx]

Supplementary Table 2. Cytokine ratios (adjusted for age and sex) relative to the Control group.

| **Cytokine** | **ICUS** | **MDS** | **CMML** |
| --- | --- | --- | --- |
| **CCL8** | 1.5 (1.1, 2) p=0.0081 | 1.9 (1.5, 2.3) p=< 1e-04 | 1.3 (1, 1.7) p=0.032 |
| **IL33** | 1.7 (0.94, 3.1) p=0.077 | 1.5 (0.88, 2.5) p=0.14 | 1.7 (1, 2.9) p=0.050 |
| **CXCL12** | 1.4 (0.98, 1.9) p=0.064 | 1.4 (1.1, 1.9) p=0.0084 | 0.97 (0.69, 1.3) p=0.83 |
| **OLR1** | 1.1 (0.81, 1.4) p=0.60 | 1.4 (1.1, 1.8) p=0.0073 | 2.5 (1.8, 3.4) p=< 1e-04 |
| **IL27** | 1.6 (1, 2.6) p=0.049 | 1.5 (0.99, 2.1) p=0.056 | 1.6 (0.95, 2.5) p=0.08 |
| **IL2** | 1 (0.57, 1.8) p=0.97 | 1.1 (0.65, 1.7) p=0.84 | 0.94 (0.55, 1.6) p=0.83 |
| **CXCL9** | 0.96 (0.67, 1.4) p=0.81 | 1 (0.73, 1.4) p=0.97 | 0.8 (0.56, 1.1) p=0.22 |
| **TGFA** | 0.96 (0.76, 1.2) p=0.75 | 1.2 (0.97, 1.4) p=0.096 | 1.5 (1.1, 1.9) p=0.0030 |
| **IL1B** | 1.3 (0.87, 1.9) p=0.20 | 1.7 (1.3, 2.3) p=4.9e-04 | 2 (1.4, 3) p=3.5e-04 |
| **IL6** | 0.69 (0.43, 1.1) p=0.10 | 1.1 (0.77, 1.7) p=0.52 | 1.2 (0.72, 1.9) p=0.55 |
| **IL4** | 0.38 (0.15, 0.99) p=0.047 | 0.54 (0.23, 1.3) p=0.16 | 0.56 (0.21, 1.5) p=0.23 |
| **TNFSF12** | 0.86 (0.75, 0.99) p=0.039 | 0.77 (0.7, 0.86) p=< 1e-04 | 0.85 (0.75, 0.96) p=0.0091 |
| **TSLP** | 0.82 (0.46, 1.5) p=0.50 | 0.87 (0.53, 1.4) p=0.60 | 0.83 (0.48, 1.4) p=0.51 |
| **CCL11** | 0.99 (0.78, 1.3) p=0.92 | 0.88 (0.74, 1) p=0.14 | 0.83 (0.68, 1) p=0.063 |
| **HGF** | 0.84 (0.68, 1) p=0.13 | 1.1 (0.91, 1.3) p=0.37 | 1.4 (1.1, 1.7) p=0.0049 |
| **FLT3LG** | 0.68 (0.49, 0.93) p=0.017 | 0.78 (0.62, 1) p=0.047 | 0.23 (0.16, 0.33) p=< 1e-04 |
| **IL17F** | 0.88 (0.49, 1.6) p=0.66 | 1.1 (0.65, 1.7) p=0.82 | 1.1 (0.65, 2) p=0.66 |
| **IL7** | 2.5 (1.6, 3.8) p=< 1e-04 | 1.9 (1.3, 2.9) p=0.0011 | 2.2 (1.5, 3.4) p=2e-04 |
| **IL13** | 0.61 (0.22, 1.7) p=0.33 | 0.69 (0.27, 1.7) p=0.43 | 0.97 (0.38, 2.5) p=0.94 |
| **IL18** | 1.1 (0.85, 1.4) p=0.51 | 1.5 (1.2, 1.9) p=1.3e-04 | 1.5 (1.2, 1.9) p=0.0012 |
| **CCL13** | 1.9 (1.4, 2.5) p=< 1e-04 | 2.2 (1.8, 2.8) p=< 1e-04 | 1.2 (0.92, 1.5) p=0.18 |
| **TNFSF10** | 0.7 (0.58, 0.83) p=1e-04 | 0.63 (0.55, 0.73) p=< 1e-04 | 0.76 (0.64, 0.91) p=0.0026 |
| **CXCL10** | 0.95 (0.54, 1.7) p=0.86 | 0.8 (0.47, 1.4) p=0.40 | 0.91 (0.51, 1.6) p=0.73 |
| **IFNG** | 1.1 (0.65, 1.7) p=0.84 | 0.97 (0.69, 1.4) p=0.85 | 0.8 (0.53, 1.2) p=0.28 |
| **IL10** | 1 (0.66, 1.5) p=0.98 | 1.6 (1.1, 2.2) p=0.0099 | 0.9 (0.6, 1.4) p=0.62 |
| **CCL19** | 0.85 (0.59, 1.2) p=0.41 | 0.74 (0.53, 1) p=0.084 | 0.88 (0.61, 1.3) p=0.49 |
| **TNF** | 0.97 (0.77, 1.2) p=0.77 | 1.2 (1, 1.4) p=0.043 | 1.2 (0.99, 1.5) p=0.068 |
| **IL15** | 0.86 (0.73, 1) p=0.073 | 1.2 (1.1, 1.3) p=0.0011 | 1 (0.89, 1.2) p=0.62 |
| **CCL3** | 1.2 (0.94, 1.5) p=0.13 | 1.9 (1.6, 2.3) p=< 1e-04 | 1.9 (1.5, 2.4) p=< 1e-04 |
| **CXCL8** | 1.4 (1, 1.8) p=0.050 | 2.5 (2, 3.1) p=< 1e-04 | 1.9 (1.3, 2.6) p=2.1e-04 |
| **MMP12** | 0.93 (0.69, 1.2) p=0.62 | 0.7 (0.55, 0.88) p=0.003 | 0.75 (0.57, 1) p=0.050 |
| **CSF2** | 0.89 (0.57, 1.4) p=0.63 | 1.2 (0.83, 1.8) p=0.32 | 0.92 (0.57, 1.5) p=0.73 |
| **CSF3** | 0.54 (0.39, 0.75) p=2.4e-04 | 0.6 (0.45, 0.79) p=3.9e-04 | 0.34 (0.23, 0.5) p=< 1e-04 |
| **VEGFA** | 1.2 (0.88, 1.5) p=0.28 | 1.3 (1.1, 1.5) p=0.01 | 1.3 (1, 1.6) p=0.021 |
| **IL17C** | 1 (0.6, 1.7) p=0.98 | 0.86 (0.57, 1.3) p=0.45 | 0.79 (0.5, 1.3) p=0.32 |
| **EGF** | 16 (8.8, 28) p=< 1e-04 | 6.8 (4, 12) p=< 1e-04 | 12 (6.9, 22) p=< 1e-04 |
| **CCL2** | 0.92 (0.74, 1.1) p=0.40 | 1.1 (1, 1.3) p=0.05 | 0.52 (0.45, 0.6) p=< 1e-04 |
| **IL17A** | 0.8 (0.35, 1.9) p=0.61 | 0.68 (0.32, 1.5) p=0.32 | 0.62 (0.28, 1.4) p=0.23 |
| **OSM** | 0.37 (0.25, 0.54) p=< 1e-04 | 0.84 (0.6, 1.2) p=0.30 | 0.86 (0.58, 1.3) p=0.45 |
| **CSF1** | 0.91 (0.79, 1) p=0.14 | 1.1 (0.97, 1.2) p=0.20 | 0.93 (0.84, 1) p=0.16 |
| **CCL4** | 1 (0.79, 1.3) p=0.95 | 1.4 (1.2, 1.7) p=7e-04 | 0.91 (0.72, 1.1) p=0.38 |
| **CXCL11** | 2 (1.4, 3) p=6.2e-04 | 3.5 (2.5, 4.9) p=< 1e-04 | 3 (2, 4.7) p=< 1e-04 |
| **LTA** | 1 (0.85, 1.2) p=0.85 | 1 (0.86, 1.2) p=0.95 | 0.9 (0.76, 1.1) p=0.24 |
| **CCL7** | 2.3 (1.7, 3) p=< 1e-04 | 2.8 (2.3, 3.5) p=< 1e-04 | 3.5 (2.6, 4.7) p=< 1e-04 |
| **MMP1** | 4.5 (3, 6.6) p=< 1e-04 | 3.8 (2.8, 5.4) p=< 1e-04 | 5.5 (3.5, 8.5) p=< 1e-04 |

Cytokine levels in cases are compared with those in healthy controls, with ratios estimated from models adjusted for age and sex. Values represent fold differences (case/control) with corresponding 95% confidence intervals (CIs). Ratios >1 indicate higher levels in cases, and ratios <1 indicate lower levels relative to controls. Given the potential differences in distributional form between groups, both unequal variance t-tests (t_test) and Mann–Whitney rank-sum tests (w_test) are reported. A Bartlett test (Bart_test) is provided to assess homogeneity of variances between groups. Where the Bartlett test indicates evidence of heterogeneity (p < 0.05), the Mann–Whitney test may be considered a more robust indicator; otherwise, the t-test provides the primary reference. To account for multiple testing across cytokines, Benjamini–Hochberg false discovery rate (FDR) adjustment has been applied to the t-test p-values (adjusted_t). These adjusted p-values provide control of the expected proportion of false discoveries under correlated testing. Confidence intervals are presented as pointwise (unadjusted) estimates and therefore do not incorporate FDR correction; they are intended to indicate effect size and precision rather than to support multiplicity-adjusted inference. All comparisons share a common control group and cytokines are biologically correlated; therefore, tests are not statistically independent. Accordingly, these analyses are interpreted as exploratory effect-size summaries, and emphasis is placed on the magnitude and consistency of observed differences rather than on individual p-values.
