# Supplementary material for: Inflammatory signatures in the spectrum of myeloid diseases
Source: Hemasphere. 2026 Jul 7;10(7):e70428. doi: 10.1002/hem3.70428 (PMC13340139; doi:10.1002/hem3.70428)
Supplement: Supplementary file 5 — Supporting Information. [file HEM3-10-e70428-s006.docx]

Supplementary Table 3. Cytokine ratios (adjusted for age and sex) relative to the ICUS group.

| **Cytokine** | **Controls** | **MDS** | **CMML** |
| --- | --- | --- | --- |
| **CCL8** | 0.68 (0.51, 0.9) p=0.0081 | 1.3 (1, 1.6) p=0.048 | 0.88 (0.68, 1.2) p=0.37 |
| **IL33** | 0.58 (0.32, 1.1) p=0.077 | 0.86 (0.59, 1.3) p=0.43 | 0.99 (0.66, 1.5) p=0.96 |
| **CXCL12** | 0.73 (0.52, 1) p=0.064 | 1 (0.82, 1.3) p=0.74 | 0.7 (0.51, 0.96) p=0.029 |
| **OLR1** | 0.93 (0.7, 1.2) p=0.60 | 1.3 (1, 1.7) p=0.048 | 2.3 (1.7, 3.2) p=< 1e-04 |
| **IL27** | 0.62 (0.38, 1) p=0.049 | 0.9 (0.64, 1.3) p=0.57 | 0.96 (0.62, 1.5) p=0.86 |
| **IL2** | 0.99 (0.56, 1.7) p=0.97 | 1 (0.74, 1.5) p=0.83 | 0.93 (0.61, 1.4) p=0.74 |
| **CXCL9** | 1 (0.74, 1.5) p=0.81 | 1.1 (0.82, 1.3) p=0.69 | 0.83 (0.62, 1.1) p=0.22 |
| **TGFA** | 1 (0.82, 1.3) p=0.75 | 1.2 (1, 1.5) p=0.054 | 1.5 (1.2, 1.9) p=9.9e-04 |
| **IL1B** | 0.77 (0.52, 1.2) p=0.20 | 1.3 (0.94, 1.8) p=0.10 | 1.6 (1, 2.4) p=0.036 |
| **IL6** | 1.5 (0.92, 2.3) p=0.10 | 1.7 (1.2, 2.3) p=0.0012 | 1.7 (1.1, 2.5) p=0.014 |
| **IL4** | 2.6 (1, 6.8) p=0.047 | 1.4 (0.82, 2.5) p=0.21 | 1.5 (0.72, 3) p=0.29 |
| **TNFSF12** | 1.2 (1, 1.3) p=0.039 | 0.9 (0.8, 1) p=0.063 | 0.98 (0.86, 1.1) p=0.78 |
| **TSLP** | 1.2 (0.68, 2.2) p=0.50 | 1.1 (0.74, 1.5) p=0.74 | 1 (0.65, 1.6) p=0.95 |
| **CCL11** | 1 (0.8, 1.3) p=0.92 | 0.89 (0.74, 1.1) p=0.22 | 0.84 (0.68, 1) p=0.10 |
| **HGF** | 1.2 (0.95, 1.5) p=0.13 | 1.3 (1.1, 1.5) p=0.003 | 1.6 (1.3, 2) p=< 1e-04 |
| **FLT3LG** | 1.5 (1.1, 2) p=0.017 | 1.2 (0.83, 1.6) p=0.38 | 0.34 (0.22, 0.51) p=< 1e-04 |
| **IL17F** | 1.1 (0.63, 2.1) p=0.66 | 1.2 (0.8, 1.8) p=0.37 | 1.3 (0.79, 2.1) p=0.31 |
| **IL7** | 0.4 (0.26, 0.61) p=< 1e-04 | 0.78 (0.57, 1.1) p=0.11 | 0.9 (0.64, 1.3) p=0.54 |
| **IL13** | 1.7 (0.6, 4.6) p=0.33 | 1.1 (0.67, 2) p=0.63 | 1.6 (0.87, 2.9) p=0.13 |
| **IL18** | 0.92 (0.72, 1.2) p=0.51 | 1.4 (1.2, 1.7) p=3.4e-04 | 1.4 (1.1, 1.7) p=0.0025 |
| **CCL13** | 0.53 (0.39, 0.73) p=< 1e-04 | 1.2 (0.92, 1.5) p=0.18 | 0.64 (0.48, 0.86) p=0.0028 |
| **TNFSF10** | 1.4 (1.2, 1.7) p=1e-04 | 0.91 (0.8, 1) p=0.15 | 1.1 (0.93, 1.3) p=0.28 |
| **CXCL10** | 1.1 (0.59, 1.9) p=0.86 | 0.84 (0.62, 1.1) p=0.24 | 0.95 (0.68, 1.3) p=0.77 |
| **IFNG** | 0.95 (0.59, 1.5) p=0.84 | 0.92 (0.63, 1.3) p=0.67 | 0.76 (0.49, 1.2) p=0.20 |
| **IL10** | 0.99 (0.65, 1.5) p=0.98 | 1.6 (1.1, 2.2) p=0.018 | 0.9 (0.58, 1.4) p=0.61 |
| **CCL19** | 1.2 (0.81, 1.7) p=0.41 | 0.87 (0.69, 1.1) p=0.23 | 1 (0.79, 1.3) p=0.83 |
| **TNF** | 1 (0.82, 1.3) p=0.77 | 1.2 (1, 1.5) p=0.019 | 1.3 (1, 1.6) p=0.032 |
| **IL15** | 1.2 (0.99, 1.4) p=0.073 | 1.4 (1.2, 1.6) p=1.3e-04 | 1.2 (0.99, 1.5) p=0.057 |
| **CCL3** | 0.83 (0.65, 1.1) p=0.13 | 1.6 (1.3, 2) p=< 1e-04 | 1.5 (1.2, 2.1) p=0.0039 |
| **CXCL8** | 0.74 (0.55, 1) p=0.050 | 1.8 (1.3, 2.5) p=3e-04 | 1.4 (0.94, 2.1) p=0.10 |
| **MMP12** | 1.1 (0.8, 1.4) p=0.62 | 0.75 (0.58, 0.96) p=0.024 | 0.81 (0.61, 1.1) p=0.16 |
| **CSF2** | 1.1 (0.71, 1.8) p=0.63 | 1.4 (1, 1.8) p=0.025 | 1 (0.69, 1.5) p=0.89 |
| **CSF3** | 1.9 (1.3, 2.6) p=2.4e-04 | 1.1 (0.88, 1.4) p=0.38 | 0.63 (0.45, 0.89) p=0.0094 |
| **VEGFA** | 0.86 (0.66, 1.1) p=0.28 | 1.1 (0.85, 1.4) p=0.52 | 1.1 (0.85, 1.5) p=0.41 |
| **IL17C** | 0.99 (0.6, 1.7) p=0.98 | 0.85 (0.59, 1.2) p=0.38 | 0.79 (0.51, 1.2) p=0.28 |
| **EGF** | 0.064 (0.036, 0.11) p=< 1e-04 | 0.43 (0.28, 0.66) p=1.3e-04 | 0.78 (0.48, 1.3) p=0.34 |
| **CCL2** | 1.1 (0.89, 1.3) p=0.40 | 1.2 (0.99, 1.5) p=0.057 | 0.57 (0.46, 0.71) p=< 1e-04 |
| **IL17A** | 1.2 (0.54, 2.9) p=0.61 | 0.85 (0.54, 1.3) p=0.49 | 0.78 (0.46, 1.3) p=0.34 |
| **OSM** | 2.7 (1.9, 4) p=< 1e-04 | 2.3 (1.7, 3) p=< 1e-04 | 2.3 (1.7, 3.3) p=< 1e-04 |
| **CSF1** | 1.1 (0.97, 1.3) p=0.14 | 1.2 (1, 1.3) p=0.0051 | 1 (0.91, 1.1) p=0.69 |
| **CCL4** | 0.99 (0.78, 1.3) p=0.95 | 1.4 (1.1, 1.8) p=0.0031 | 0.9 (0.7, 1.1) p=0.39 |
| **CXCL11** | 0.5 (0.34, 0.74) p=6.2e-04 | 1.7 (1.2, 2.5) p=0.0018 | 1.5 (1, 2.3) p=0.045 |
| **LTA** | 0.98 (0.82, 1.2) p=0.85 | 0.99 (0.88, 1.1) p=0.84 | 0.89 (0.78, 1) p=0.072 |
| **CCL7** | 0.44 (0.33, 0.58) p=< 1e-04 | 1.2 (0.94, 1.6) p=0.13 | 1.5 (1.1, 2.1) p=0.016 |
| **MMP1** | 0.22 (0.15, 0.33) p=< 1e-04 | 0.86 (0.59, 1.3) p=0.45 | 1.2 (0.75, 2) p=0.41 |

Cytokine levels in healthy controls and cases are compared with those in ICUS, with ratios estimated from models adjusted for age and sex. Values represent fold differences with corresponding 95% confidence intervals (CIs). Ratios >1 indicate higher levels in cases, and ratios <1 indicate lower levels relative to ICUS group. Given the potential differences in distributional form between groups, both unequal variance t-tests (t_test) and Mann–Whitney rank-sum tests (w_test) are reported. A Bartlett test (Bart_test) is provided to assess homogeneity of variances between groups. Where the Bartlett test indicates evidence of heterogeneity (p < 0.05), the Mann–Whitney test may be considered a more robust indicator; otherwise, the t-test provides the primary reference. No correction for multiple testing was applied. Confidence intervals are presented as pointwise (unadjusted) estimates and therefore do not incorporate FDR correction; they are intended to indicate effect size and precision rather than to support multiplicity-adjusted inference. All comparisons share a common control group (ICUS) and cytokines are biologically correlated; therefore, tests are not statistically independent. Accordingly, these analyses are interpreted as exploratory effect-size summaries, and emphasis is placed on the magnitude and consistency of observed differences rather than on individual p-values.
