# Supplementary material for: Inflammatory signatures in the spectrum of myeloid diseases
Source: Hemasphere. 2026 Jul 7;10(7):e70428. doi: 10.1002/hem3.70428 (PMC13340139; doi:10.1002/hem3.70428)
Supplement: Supplementary file 7 — Supporting Information. [file HEM3-10-e70428-s012.docx]

Supplementary Table 5. Cytokine ratios (adjusted for age and sex) relative to the CMML group.

| **Cytokine** | **Controls** | **ICUS** | **MDS** |
| --- | --- | --- | --- |
| **CCL8** | 0.77 (0.6, 0.98) p=0.032 | 1.1 (0.86, 1.5) p=0.37 | 1.4 (1.2, 1.7) p=< 1e-04 |
| **IL33** | 0.59 (0.34, 1) p=0.05 | 1 (0.68, 1.5) p=0.96 | 0.87 (0.68, 1.1) p=0.25 |
| **CXCL12** | 1 (0.74, 1.4) p=0.83 | 1.4 (1, 1.9) p=0.029 | 1.5 (1.2, 1.9) p=0.0015 |
| **OLR1** | 0.4 (0.29, 0.56) p=< 1e-04 | 0.44 (0.31, 0.6) p=< 1e-04 | 0.57 (0.42, 0.76) p=2.2e-04 |
| **IL27** | 0.64 (0.39, 1.1) p=0.08 | 1 (0.67, 1.6) p=0.86 | 0.94 (0.66, 1.3) p=0.73 |
| **IL2** | 1.1 (0.62, 1.8) p=0.83 | 1.1 (0.71, 1.6) p=0.74 | 1.1 (0.82, 1.5) p=0.49 |
| **CXCL9** | 1.3 (0.87, 1.8) p=0.22 | 1.2 (0.9, 1.6) p=0.22 | 1.3 (0.98, 1.6) p=0.072 |
| **TGFA** | 0.69 (0.54, 0.88) p=0.003 | 0.66 (0.52, 0.85) p=9.9e-04 | 0.8 (0.66, 0.98) p=0.032 |
| **IL1B** | 0.5 (0.34, 0.73) p=3.5e-04 | 0.64 (0.42, 0.97) p=0.036 | 0.85 (0.62, 1.2) p=0.30 |
| **IL6** | 0.87 (0.54, 1.4) p=0.55 | 0.59 (0.39, 0.9) p=0.014 | 0.99 (0.7, 1.4) p=0.93 |
| **IL4** | 1.8 (0.69, 4.7) p=0.23 | 0.68 (0.33, 1.4) p=0.29 | 0.97 (0.55, 1.7) p=0.90 |
| **TNFSF12** | 1.2 (1, 1.3) p=0.0091 | 1 (0.89, 1.2) p=0.78 | 0.91 (0.83, 1) p=0.063 |
| **TSLP** | 1.2 (0.69, 2.1) p=0.51 | 0.99 (0.63, 1.5) p=0.95 | 1.1 (0.75, 1.5) p=0.78 |
| **CCL11** | 1.2 (0.99, 1.5) p=0.063 | 1.2 (0.96, 1.5) p=0.10 | 1.1 (0.92, 1.2) p=0.43 |
| **HGF** | 0.74 (0.6, 0.91) p=0.0049 | 0.62 (0.51, 0.76) p=< 1e-04 | 0.8 (0.69, 0.93) p=0.0042 |
| **FLT3LG** | 4.4 (3.1, 6.3) p=< 1e-04 | 3 (2, 4.5) p=< 1e-04 | 3.4 (2.4, 4.9) p=< 1e-04 |
| **IL17F** | 0.88 (0.5, 1.5) p=0.66 | 0.77 (0.47, 1.3) p=0.31 | 0.93 (0.65, 1.4) p=0.72 |
| **IL7** | 0.45 (0.29, 0.68) p=2e-04 | 1.1 (0.79, 1.6) p=0.54 | 0.87 (0.64, 1.2) p=0.36 |
| **IL13** | 1 (0.4, 2.7) p=0.94 | 0.63 (0.34, 1.1) p=0.13 | 0.71 (0.46, 1.1) p=0.13 |
| **IL18** | 0.67 (0.52, 0.85) p=0.0012 | 0.72 (0.59, 0.89) p=0.0025 | 1 (0.85, 1.2) p=0.88 |
| **CCL13** | 0.84 (0.65, 1.1) p=0.18 | 1.6 (1.2, 2.1) p=0.0028 | 1.9 (1.5, 2.3) p=< 1e-04 |
| **TNFSF10** | 1.3 (1.1, 1.6) p=0.0026 | 0.91 (0.78, 1.1) p=0.28 | 0.83 (0.74, 0.94) p=0.0043 |
| **CXCL10** | 1.1 (0.63, 1.9) p=0.73 | 1.1 (0.75, 1.5) p=0.77 | 0.88 (0.67, 1.2) p=0.36 |
| **IFNG** | 1.3 (0.83, 1.9) p=0.28 | 1.3 (0.86, 2) p=0.20 | 1.2 (0.91, 1.6) p=0.19 |
| **IL10** | 1.1 (0.74, 1.7) p=0.62 | 1.1 (0.73, 1.7) p=0.61 | 1.7 (1.2, 2.5) p=0.0029 |
| **CCL19** | 1.1 (0.79, 1.6) p=0.49 | 0.97 (0.74, 1.3) p=0.83 | 0.84 (0.68, 1.1) p=0.14 |
| **TNF** | 0.82 (0.66, 1) p=0.068 | 0.79 (0.64, 0.98) p=0.032 | 0.98 (0.84, 1.2) p=0.83 |
| **IL15** | 0.96 (0.82, 1.1) p=0.62 | 0.83 (0.68, 1) p=0.057 | 1.1 (0.98, 1.3) p=0.10 |
| **CCL3** | 0.54 (0.42, 0.68) p=< 1e-04 | 0.65 (0.48, 0.87) p=0.0039 | 1 (0.82, 1.3) p=0.75 |
| **CXCL8** | 0.53 (0.38, 0.74) p=2.1e-04 | 0.72 (0.49, 1.1) p=0.10 | 1.3 (0.92, 1.9) p=0.13 |
| **MMP12** | 1.3 (1, 1.8) p=0.050 | 1.2 (0.92, 1.6) p=0.16 | 0.92 (0.73, 1.2) p=0.48 |
| **CSF2** | 1.1 (0.68, 1.8) p=0.73 | 0.97 (0.65, 1.4) p=0.89 | 1.3 (0.95, 1.8) p=0.096 |
| **CSF3** | 2.9 (2, 4.3) p=< 1e-04 | 1.6 (1.1, 2.2) p=0.0094 | 1.7 (1.3, 2.4) p=5.7e-04 |
| **VEGFA** | 0.77 (0.62, 0.96) p=0.021 | 0.89 (0.68, 1.2) p=0.41 | 0.97 (0.79, 1.2) p=0.73 |
| **IL17C** | 1.3 (0.79, 2) p=0.32 | 1.3 (0.82, 2) p=0.28 | 1.1 (0.81, 1.4) p=0.59 |
| **EGF** | 0.081 (0.046, 0.15) p=< 1e-04 | 1.3 (0.77, 2.1) p=0.34 | 0.55 (0.36, 0.86) p=0.0081 |
| **CCL2** | 1.9 (1.7, 2.2) p=< 1e-04 | 1.8 (1.4, 2.2) p=< 1e-04 | 2.2 (1.9, 2.5) p=< 1e-04 |
| **IL17A** | 1.6 (0.73, 3.5) p=0.23 | 1.3 (0.76, 2.2) p=0.34 | 1.1 (0.77, 1.6) p=0.60 |
| **OSM** | 1.2 (0.78, 1.7) p=0.45 | 0.43 (0.3, 0.6) p=< 1e-04 | 0.98 (0.72, 1.3) p=0.87 |
| **CSF1** | 1.1 (0.97, 1.2) p=0.16 | 0.98 (0.87, 1.1) p=0.69 | 1.1 (1.1, 1.2) p=2.6e-04 |
| **CCL4** | 1.1 (0.88, 1.4) p=0.38 | 1.1 (0.87, 1.4) p=0.39 | 1.6 (1.3, 1.9) p=< 1e-04 |
| **CXCL11** | 0.33 (0.21, 0.51) p=< 1e-04 | 0.66 (0.44, 0.99) p=0.045 | 1.2 (0.79, 1.7) p=0.47 |
| **LTA** | 1.1 (0.93, 1.3) p=0.24 | 1.1 (0.99, 1.3) p=0.072 | 1.1 (1, 1.2) p=0.035 |
| **CCL7** | 0.29 (0.21, 0.39) p=< 1e-04 | 0.66 (0.47, 0.92) p=0.016 | 0.8 (0.6, 1.1) p=0.15 |
| **MMP1** | 0.18 (0.12, 0.29) p=< 1e-04 | 0.82 (0.5, 1.3) p=0.41 | 0.71 (0.46, 1.1) p=0.12 |

Cytokine levels in healthy controls and cases are compared with those in CMML group, with ratios estimated from models adjusted for age and sex. Values represent fold differences with corresponding 95% confidence intervals (CIs). Ratios >1 indicate higher levels in cases, and ratios <1 indicate lower levels relative to ICUS group. Given the potential differences in distributional form between groups, both unequal variance t-tests (t_test) and Mann–Whitney rank-sum tests (w_test) are reported. A Bartlett test (Bart_test) is provided to assess homogeneity of variances between groups. Where the Bartlett test indicates evidence of heterogeneity (p < 0.05), the Mann–Whitney test may be considered a more robust indicator; otherwise, the t-test provides the primary reference. No correction for multiple testing was applied. Confidence intervals are presented as pointwise (unadjusted) estimates and therefore do not incorporate FDR correction; they are intended to indicate effect size and precision rather than to support multiplicity-adjusted inference. All comparisons share a common control group (CMLL) and cytokines are biologically correlated; therefore, tests are not statistically independent. Accordingly, these analyses are interpreted as exploratory effect-size summaries, and emphasis is placed on the magnitude and consistency of observed differences rather than on individual p-values.
