# Supplementary material for: Inflammatory signatures in the spectrum of myeloid diseases
Source: Hemasphere. 2026 Jul 7;10(7):e70428. doi: 10.1002/hem3.70428 (PMC13340139; doi:10.1002/hem3.70428)
Supplement: Supplementary file 8 — Supporting Information. [file HEM3-10-e70428-s011.docx]

Supplementary Table 6.

|  | **Chromatin** | **Cohesin Complex** | **DNA Methylation** | **RNA Splicing** | **Signalling** | **Transcription** | **Tumor Suppressor** |
| --- | --- | --- | --- | --- | --- | --- | --- |
| **CCL8** | 0.04 | 0.11 | 0.04 | 0.07 | -0.09 | -0.08 | 0.04 |
| **IL33** | -0.02 | -0.06 | 0.05 | 0.09 | 0.03 | 0.06 | 0.12(.) |
| **CXCL12** | -0.09 | 0.02 | -0.2(*) | -0.17(.) | -0.04 | -0.23(*) | 0 |
| **OLR1** | 0.05 | -0.09 | 0.01 | -0.02 | 0.19(**) | -0.07 | 0.08 |
| **IL27** | 0.12(.) | 0.08 | 0.04 | 0.02 | 0.2(**) | 0.07 | -0.02 |
| **IL2** | 0.02 | 0 | -0.05 | 0.08 | 0.02 | -0.03 | 0.09 |
| **CXCL9** | -0.06 | 0.19(**) | 0 | 0.03 | 0.01 | -0.05 | -0.07 |
| **TGFA** | 0.07 | -0.04 | 0.05 | -0.03 | 0.12(.) | -0.04 | 0.04 |
| **IL1B** | 0.2(**) | 0.17(*) | 0.13(.) | 0.1 | 0.14(*) | 0.16(*) | 0.14(*) |
| **IL6** | 0.13(*) | 0.05 | 0.11(.) | 0.03 | 0.16(*) | 0.03 | 0.03 |
| **IL4** | 0 | 0.09 | 0.03 | 0.13 | 0.13 | 0.06 | 0.13 |
| **TNFSF12** | -0.02 | -0.02 | 0.08 | 0.11(.) | 0.03 | -0.03 | -0.04 |
| **TSLP** | 0.08 | 0.29(***) | -0.08 | -0.04 | 0 | 0.1 | 0 |
| **CCL11** | 0.01 | 0.04 | 0.08 | 0.22(***) | -0.21(**) | -0.06 | -0.06 |
| **HGF** | 0.17(*) | -0.01 | 0.09 | 0.12(.) | 0.18(**) | 0.04 | 0.11 |
| **FLT3LG** | -0.05 | 0.02 | -0.31(***) | -0.22(**) | -0.29(***) | -0.22(**) | -0.06 |
| **IL17F** | 0.07 | 0.01 | 0 | 0.07 | 0.01 | 0.01 | 0.09 |
| **IL7** | -0.06 | -0.24(***) | 0.11 | -0.12(.) | -0.02 | -0.17(*) | -0.12(.) |
| **IL13** | -0.04 | 0 | 0.01 | 0.1 | 0.1 | 0.02 | 0.09 |
| **IL18** | 0 | 0.09 | 0.08 | 0.12(.) | 0.19(**) | 0.14(*) | 0.07 |
| **CCL13** | 0.01 | 0.11(.) | -0.08 | 0.01 | -0.15(*) | -0.07 | 0.03 |
| **TNFSF10** | -0.05 | -0.08 | 0.04 | -0.02 | 0.17(*) | 0 | -0.04 |
| **CXCL10** | -0.07 | 0.1 | 0.06 | 0.01 | -0.04 | -0.09 | -0.06 |
| **IFNG** | -0.06 | 0.02 | -0.04 | -0.05 | -0.07 | -0.17(*) | 0.09 |
| **IL10** | 0.01 | 0.23(***) | -0.16(*) | -0.06 | 0.13(.) | 0.11(.) | 0.07 |
| **CCL19** | 0.02 | 0.09 | 0.06 | 0.16(*) | 0.03 | -0.03 | -0.04 |
| **TNF** | 0.05 | 0.08 | -0.02 | -0.01 | 0.17(*) | 0.01 | 0.13(.) |
| **IL15** | 0.17(*) | 0.08 | 0.03 | -0.01 | 0.12(.) | 0.07 | 0.14(*) |
| **CCL3** | 0.16(*) | 0.14(*) | 0.06 | 0.08 | 0.11(.) | 0.08 | 0.07 |
| **CXCL8** | 0.29(***) | 0.45(***) | 0 | 0.2(**) | 0.05 | 0.24(***) | 0.19(**) |
| **MMP12** | -0.02 | 0.11 | -0.03 | 0.18(**) | 0.04 | 0.05 | 0.09 |
| **CSF2** | 0.09 | 0.18(**) | -0.09 | -0.01 | -0.08 | 0 | 0.1 |
| **CSF3** | -0.04 | -0.04 | -0.21(**) | -0.05 | -0.17(*) | -0.09 | 0.06 |
| **VEGFA** | 0.15(*) | 0.08 | 0.05 | 0.03 | 0.15(*) | -0.05 | -0.04 |
| **IL17C** | 0.08 | 0.04 | -0.08 | -0.04 | 0.08 | -0.07 | 0.01 |
| **EGF** | -0.07 | -0.06 | 0.08 | -0.06 | -0.02 | -0.05 | -0.09 |
| **CCL2** | 0.01 | 0.26(***) | -0.27(***) | -0.03 | -0.18(**) | 0 | 0.09 |
| **IL17A** | 0.04 | 0.1 | -0.08 | 0.05 | -0.05 | -0.12(.) | 0.1 |
| **OSM** | 0.09 | -0.04 | -0.05 | -0.22(**) | 0.14(*) | -0.09 | 0.21(**) |
| **CSF1** | 0.02 | 0.07 | -0.02 | -0.19(**) | 0.09 | -0.09 | 0.07 |
| **CCL4** | 0.18(**) | 0.12(.) | -0.12(.) | 0.06 | -0.04 | 0.03 | 0.16(*) |
| **CXCL11** | 0.02 | 0.15(*) | 0.04 | -0.13(.) | 0.17(*) | 0.09 | -0.03 |
| **LTA** | -0.01 | 0.07 | -0.16(*) | -0.1 | 0.02 | -0.02 | -0.02 |
| **CCL7** | -0.03 | 0.04 | 0.27(***) | 0.27(***) | 0.04 | -0.02 | -0.04 |
| **MMP1** | -0.09 | -0.16(*) | 0.1 | -0.19(**) | 0.01 | -0.25(***) | -0.16(*) |

Pearson correlation coefficients (with p-value codes) for the correlation between mutation pathways and cytokine levels. (N.B. This corresponds to considering cytokine levels as outcome, and mutation pathway as explanatory, in linear regression. Therefore, the correlation coefficient corresponds to the square root of the explained variation R^2^.)

**p-value codes**

[0, 0.001] = (***)

(0.001, 0.01] = (**)

(0.01, 0.05] = (*)

(0.05, 0.1] = (.)
