# Supplementary material for: Inflammatory signatures in the spectrum of myeloid diseases
Source: Hemasphere. 2026 Jul 7;10(7):e70428. doi: 10.1002/hem3.70428 (PMC13340139; doi:10.1002/hem3.70428)
Supplement: Supplementary file 9 — Supporting Information. [file HEM3-10-e70428-s009.docx]

Supplementary Table 7

|  | **ASXL1** | **CBL** | **DNMT3A** | **JAK2** | **RUNX1** | **SF3B1** | **SRSF2** | **TET2** | **U2AF1** | **ZRSR2** |
| --- | --- | --- | --- | --- | --- | --- | --- | --- | --- | --- |
| **CCL8** | 0.06 | -0.12(.) | 0.04 | 0.02 | -0.07 | 0.07 | -0.1 | 0.03 | 0.13(*) | 0.13(.) |
| **IL33** | 0.03 | 0.04 | -0.08 | 0.02 | 0.06 | -0.04 | 0.09 | 0.06 | 0.01 | 0.01 |
| **CXCL12** | -0.11 | -0.05 | 0 | 0.03 | -0.23(*) | 0.29(**) | -0.12 | -0.2(*) | -0.08 | -0.02 |
| **OLR1** | 0.09 | 0.02 | -0.06 | 0.09 | -0.03 | 0.09 | 0.01 | 0.05 | 0.1 | -0.15(*) |
| **IL27** | 0.1 | 0.02 | -0.13(.) | 0.09 | -0.04 | -0.04 | 0.06 | 0.1 | -0.13(.) | 0.05 |
| **IL2** | 0.03 | 0.07 | -0.09 | 0.08 | 0.05 | -0.05 | 0.08 | 0.04 | 0.04 | 0 |
| **CXCL9** | -0.04 | -0.04 | -0.09 | 0.12(.) | -0.03 | -0.07 | -0.02 | 0.12(.) | -0.06 | 0.1 |
| **TGFA** | 0.08 | -0.03 | -0.06 | 0.15(*) | -0.05 | 0.03 | -0.01 | 0.15(*) | 0.04 | -0.08 |
| **IL1B** | 0.2(**) | 0.04 | 0.07 | 0.11(.) | 0.26(***) | -0.03 | 0.09 | -0.02 | 0.06 | -0.03 |
| **IL6** | 0.1 | 0.01 | 0.1 | 0.02 | 0.07 | -0.02 | -0.06 | 0.06 | 0.06 | 0 |
| **IL4** | 0 | 0 | -0.08 | 0.24(**) | 0.11 | 0.1 | 0.13 | 0.14 | -0.08 | 0.09 |
| **TNFSF12** | -0.04 | -0.04 | -0.13(.) | 0.06 | -0.1 | -0.1 | 0.14(*) | 0.13(.) | -0.02 | 0.1 |
| **TSLP** | 0.08 | 0 | -0.13(.) | 0.07 | 0.15(*) | 0.02 | 0.07 | -0.06 | -0.08 | -0.11 |
| **CCL11** | 0.07 | -0.02 | -0.11(.) | -0.1 | -0.07 | -0.22(***) | 0.11 | 0.16(*) | 0.06 | 0.18(**) |
| **HGF** | 0.18(**) | -0.01 | -0.06 | 0.15(*) | 0.09 | 0.02 | 0.1 | 0.08 | 0.09 | -0.04 |
| **FLT3LG** | -0.03 | -0.19(**) | 0.01 | 0 | -0.19(**) | 0.15(*) | -0.27(***) | -0.29(***) | 0.06 | -0.04 |
| **IL17F** | 0.06 | 0 | -0.02 | 0.02 | -0.04 | -0.03 | 0.05 | 0.01 | 0.03 | 0 |
| **IL7** | -0.06 | -0.14(*) | 0.04 | 0.13(.) | -0.21(**) | 0.34(***) | -0.15(*) | 0.03 | 0 | 0.04 |
| **IL13** | -0.06 | 0.08 | -0.05 | 0.11(.) | 0.07 | -0.04 | 0.15(*) | 0.07 | 0 | -0.03 |
| **IL18** | 0.02 | -0.01 | -0.03 | 0.13(.) | 0.13(*) | -0.11 | 0.13(*) | 0.04 | 0.08 | -0.06 |
| **CCL13** | 0 | -0.16(*) | 0.01 | -0.02 | -0.05 | 0.05 | -0.1 | -0.06 | 0.07 | 0.08 |
| **TNFSF10** | -0.05 | -0.15(*) | 0.09 | 0.07 | -0.12(.) | -0.08 | 0.05 | 0.09 | -0.06 | -0.04 |
| **CXCL10** | -0.08 | -0.14(*) | -0.04 | 0.08 | -0.04 | -0.16(*) | 0 | 0.12(.) | -0.07 | 0.07 |
| **IFNG** | -0.03 | -0.03 | -0.12(.) | 0.04 | -0.03 | -0.02 | -0.1 | 0.04 | -0.05 | 0.09 |
| **IL10** | 0.02 | -0.06 | -0.05 | 0.08 | 0.26(***) | -0.17(*) | -0.02 | -0.22(***) | -0.02 | -0.06 |
| **CCL19** | 0.02 | -0.03 | -0.06 | 0.02 | 0.07 | -0.18(**) | 0.12(.) | 0.04 | 0 | 0.11 |
| **TNF** | 0 | -0.01 | 0.03 | 0.11 | 0.07 | -0.01 | 0 | 0.07 | -0.01 | -0.06 |
| **IL15** | 0.16(*) | 0.01 | 0.07 | 0.11 | 0.14(*) | -0.05 | -0.01 | 0.02 | 0.03 | -0.12(.) |
| **CCL3** | 0.11 | -0.01 | 0.06 | 0.11(.) | 0.17(*) | -0.06 | 0.1 | 0 | 0.05 | -0.07 |
| **CXCL8** | 0.27(***) | 0.01 | 0.01 | 0.01 | 0.31(***) | -0.13(*) | 0.2(**) | -0.07 | 0.05 | -0.02 |
| **MMP12** | 0.01 | 0 | -0.13(.) | 0 | -0.01 | -0.27(***) | 0.19(**) | 0.1 | -0.03 | 0.04 |
| **CSF2** | 0.1 | -0.07 | -0.04 | 0.03 | 0.09 | 0.09 | -0.08 | -0.1 | 0.09 | -0.02 |
| **CSF3** | -0.02 | -0.06 | -0.07 | -0.26(***) | 0.01 | -0.04 | -0.14(*) | -0.29(***) | 0.02 | 0.1 |
| **VEGFA** | 0.13(*) | -0.03 | -0.04 | 0.23(***) | -0.06 | 0.08 | 0.11 | 0.08 | -0.06 | -0.04 |
| **IL17C** | 0.08 | 0.07 | -0.05 | 0 | 0.03 | -0.06 | 0.04 | 0.07 | -0.06 | -0.08 |
| **EGF** | -0.08 | -0.08 | 0 | 0.06 | -0.05 | 0.16(*) | -0.05 | 0.04 | -0.03 | 0.03 |
| **CCL2** | 0.02 | -0.18(**) | 0 | 0 | 0.02 | -0.06 | -0.13(.) | -0.23(***) | 0.06 | 0.04 |
| **IL17A** | 0.08 | 0.05 | -0.18(**) | -0.05 | 0.05 | -0.04 | 0 | 0.03 | 0.01 | 0.08 |
| **OSM** | 0.09 | -0.05 | 0.02 | 0.21(**) | 0 | 0.29(***) | -0.17(**) | -0.08 | 0.05 | -0.2(**) |
| **CSF1** | 0.01 | -0.08 | 0.04 | 0.11(.) | 0.03 | 0.2(**) | -0.14(*) | -0.02 | -0.09 | -0.06 |
| **CCL4** | 0.15(*) | -0.1 | 0.05 | 0.04 | 0.08 | -0.11(.) | -0.02 | -0.14(*) | 0.1 | 0.04 |
| **CXCL11** | 0 | -0.05 | 0.02 | 0.17(*) | 0.17(*) | 0.2(**) | -0.21(**) | 0.04 | -0.03 | 0.1 |
| **LTA** | 0.01 | 0 | -0.03 | 0.06 | 0.05 | -0.05 | 0.01 | -0.11(.) | -0.09 | -0.1 |
| **CCL7** | -0.03 | 0.07 | -0.06 | 0.08 | -0.04 | -0.07 | 0.31(***) | 0.36(***) | -0.15(*) | 0.13(*) |
| **MMP1** | -0.15(*) | 0.02 | 0.06 | 0 | -0.18(*) | 0.31(***) | -0.22(**) | 0.11 | -0.17(*) | 0.16(*) |

Pearson correlation coefficients (with p-value codes) for the correlation between mutations and cytokine levels. (N.B. This corresponds to considering cytokine levels as outcome, and mutation as explanatory, in linear regression. Therefore, the correlation coefficient corresponds to the square root of the explained variation R^2^.)

**p-value codes**

[0, 0.001] = (***)

(0.001, 0.01] = (**)

(0.01, 0.05] = (*)

(0.05, 0.1] = (.)
