# Supplementary material for: Inflammatory signatures in the spectrum of myeloid diseases
Source: Hemasphere. 2026 Jul 7;10(7):e70428. doi: 10.1002/hem3.70428 (PMC13340139; doi:10.1002/hem3.70428)
Supplement: Supplementary file 10 — Supporting Information. [file HEM3-10-e70428-s010.docx]

*Supplementary Table 8. Cytokine predictors of progression to MDS*

| variable_name | hazard_ratio | CI_lower | CI_upper | p_value | p_adj |
| --- | --- | --- | --- | --- | --- |
| CCL8 | 0.78 | 0.51 | 1.2 | 0.28 | 0.63 |
| IL33 | 1.1 | 0.87 | 1.5 | 0.33 | 0.63 |
| CXCL12 | 0.69 | 0.33 | 1.4 | 0.32 | 0.63 |
| OLR1 | 0.86 | 0.65 | 1.1 | 0.3 | 0.63 |
| IL27 | 1.2 | 0.89 | 1.6 | 0.26 | 0.63 |
| IL2 | 0.92 | 0.69 | 1.2 | 0.6 | 0.80 |
| CXCL9 | 0.67 | 0.46 | 0.99 | 0.042 | 0.63 |
| TGFA | 0.78 | 0.49 | 1.3 | 0.3 | 0.63 |
| IL1B | 1.2 | 0.93 | 1.6 | 0.15 | 0.63 |
| IL6 | 0.86 | 0.64 | 1.2 | 0.33 | 0.63 |
| IL4 | 0.79 | 0.56 | 1.1 | 0.17 | 0.63 |
| TNFSF12 | 0.98 | 0.42 | 2.3 | 0.96 | 0.98 |
| TSLP | 1.1 | 0.8 | 1.5 | 0.61 | 0.80 |
| CCL11 | 0.93 | 0.52 | 1.6 | 0.8 | 0.98 |
| HGF | 1 | 0.63 | 1.7 | 0.88 | 0.98 |
| FLT3LG | 0.73 | 0.59 | 0.92 | 0.0061 | 0.28 |
| IL17F | 1.1 | 0.91 | 1.4 | 0.26 | 0.63 |
| IL7 | 1.2 | 0.91 | 1.5 | 0.21 | 0.63 |
| IL13 | 0.95 | 0.79 | 1.1 | 0.58 | 0.80 |
| IL18 | 1.1 | 0.71 | 1.8 | 0.6 | 0.80 |
| CCL13 | 0.98 | 0.68 | 1.4 | 0.91 | 0.98 |
| TNFSF10 | 1 | 0.48 | 2.2 | 0.93 | 0.98 |
| CXCL10 | 0.82 | 0.59 | 1.1 | 0.24 | 0.63 |
| IFNG | 0.84 | 0.61 | 1.1 | 0.26 | 0.63 |
| IL10 | 0.98 | 0.76 | 1.2 | 0.85 | 0.98 |
| CCL19 | 1 | 0.66 | 1.5 | 0.98 | 0.98 |
| TNF | 0.96 | 0.55 | 1.7 | 0.89 | 0.98 |
| IL15 | 0.83 | 0.45 | 1.5 | 0.56 | 0.80 |
| CCL3 | 1 | 0.72 | 1.4 | 0.97 | 0.98 |
| CXCL8 | 0.93 | 0.73 | 1.2 | 0.59 | 0.80 |
| MMP12 | 1.1 | 0.78 | 1.6 | 0.51 | 0.80 |
| CSF2 | 0.86 | 0.61 | 1.2 | 0.4 | 0.69 |
| CSF3 | 1.1 | 0.77 | 1.6 | 0.59 | 0.80 |
| VEGFA | 1.4 | 0.94 | 2 | 0.1 | 0.63 |
| IL17C | 1 | 0.78 | 1.4 | 0.79 | 0.98 |
| EGF | 1.1 | 0.93 | 1.3 | 0.26 | 0.63 |
| CCL2 | 0.68 | 0.43 | 1.1 | 0.097 | 0.63 |
| IL17A | 1.1 | 0.89 | 1.4 | 0.39 | 0.69 |
| OSM | 0.85 | 0.65 | 1.1 | 0.23 | 0.63 |
| CSF1 | 0.9 | 0.33 | 2.4 | 0.83 | 0.98 |
| CCL4 | 1.2 | 0.88 | 1.7 | 0.23 | 0.63 |
| CXCL11 | 1.2 | 0.95 | 1.5 | 0.13 | 0.63 |
| LTA | 0.58 | 0.27 | 1.3 | 0.16 | 0.63 |
| CCL7 | 0.82 | 0.6 | 1.1 | 0.22 | 0.63 |
| MMP1 | 1.1 | 0.93 | 1.4 | 0.2 | 0.63 |

Each of these analyses is a Cox regression on the individual log cytokine value, adjusted for age and sex.
