# Supplementary material for: Inflammatory signatures in the spectrum of myeloid diseases
Source: Hemasphere. 2026 Jul 7;10(7):e70428. doi: 10.1002/hem3.70428 (PMC13340139; doi:10.1002/hem3.70428)
Supplement: Supplementary file 11 — Supporting Information. [file HEM3-10-e70428-s002.docx]

*Supplementary Table 9. Association of cytokine levels and progression to AML*

| **variable_name** | **hazard_ratio** | **CI_lower** | **CI_upper** | **p_value** | **p_adj** |
| --- | --- | --- | --- | --- | --- |
| CCL8 | 0.76 | 0.42 | 1.4 | 0.36 | 0.5900 |
| IL33 | 0.92 | 0.62 | 1.4 | 0.67 | 0.7800 |
| CXCL12 | 0.51 | 0.2 | 1.3 | 0.16 | 0.4700 |
| OLR1 | 1.1 | 0.75 | 1.5 | 0.74 | 0.7900 |
| IL27 | 1.2 | 0.83 | 1.7 | 0.34 | 0.5900 |
| IL2 | 0.83 | 0.57 | 1.2 | 0.32 | 0.5900 |
| CXCL9 | 0.78 | 0.49 | 1.2 | 0.29 | 0.5900 |
| TGFA | 0.84 | 0.45 | 1.6 | 0.57 | 0.7200 |
| IL1B | 1.3 | 0.92 | 1.7 | 0.14 | 0.4600 |
| IL6 | 1.1 | 0.86 | 1.5 | 0.37 | 0.5900 |
| IL4 | 0.85 | 0.56 | 1.3 | 0.46 | 0.6600 |
| TNFSF12 | 0.48 | 0.16 | 1.4 | 0.19 | 0.4800 |
| TSLP | 0.79 | 0.51 | 1.2 | 0.3 | 0.5900 |
| CCL11 | 0.81 | 0.38 | 1.7 | 0.59 | 0.7200 |
| HGF | 1.4 | 0.77 | 2.6 | 0.27 | 0.5900 |
| FLT3LG | 0.76 | 0.56 | 1.0 | 0.066 | 0.3300 |
| IL17F | 1.1 | 0.81 | 1.5 | 0.56 | 0.7200 |
| IL7 | 0.69 | 0.49 | 0.97 | 0.033 | 0.2100 |
| IL13 | 0.98 | 0.77 | 1.3 | 0.88 | 0.9000 |
| IL18 | 1.6 | 0.9 | 2.9 | 0.11 | 0.4200 |
| CCL13 | 0.96 | 0.59 | 1.6 | 0.88 | 0.9000 |
| TNFSF10 | 0.42 | 0.14 | 1.2 | 0.11 | 0.4200 |
| CXCL10 | 0.83 | 0.53 | 1.3 | 0.41 | 0.6100 |
| IFNG | 0.74 | 0.48 | 1.2 | 0.19 | 0.4800 |
| IL10 | 1.4 | 1.1 | 1.8 | 0.0074 | 0.0670 |
| CCL19 | 0.82 | 0.48 | 1.4 | 0.49 | 0.6600 |
| TNF | 1.4 | 0.69 | 2.9 | 0.35 | 0.5900 |
| **IL15** | **4.9** | **2.2** | **11** | **7.7e-05** | **0.0017** |
| CCL3 | 1.4 | 0.93 | 2.1 | 0.11 | 0.4200 |
| CXCL8 | 1.4 | 1.1 | 1.9 | 0.0056 | 0.0630 |
| MMP12 | 0.91 | 0.58 | 1.4 | 0.69 | 0.7800 |
| CSF2 | 1.1 | 0.73 | 1.8 | 0.57 | 0.7200 |
| CSF3 | 1.8 | 1.1 | 3 | 0.027 | 0.2000 |
| VEGFA | 0.52 | 0.27 | 1 | 0.053 | 0.3000 |
| IL17C | 1 | 0.69 | 1.5 | 0.95 | 0.9500 |
| EGF | 0.76 | 0.62 | 0.92 | 0.0055 | 0.0630 |
| CCL2 | 1.4 | 0.82 | 2.2 | 0.23 | 0.5500 |
| IL17A | 0.93 | 0.69 | 1.3 | 0.64 | 0.7600 |
| OSM | 1.3 | 0.92 | 1.8 | 0.14 | 0.4600 |
| CSF1 | 1.6 | 0.43 | 6.1 | 0.47 | 0.6600 |
| CCL4 | 1.2 | 0.79 | 1.8 | 0.39 | 0.6100 |
| CXCL11 | 0.84 | 0.6 | 1.2 | 0.29 | 0.5900 |
| LTA | 1.2 | 0.44 | 3.2 | 0.73 | 0.7900 |
| CCL7 | 0.74 | 0.48 | 1.2 | 0.18 | 0.4800 |
| **MMP1** | **0.55** | **0.41** | **0.74** | **6.6e-05** | **0.0017** |

Each of these analyses is a Cox regression on the individual log cytokine value, adjusted for age and sex.
