# Supplementary material for: Inflammatory signatures in the spectrum of myeloid diseases
Source: Hemasphere. 2026 Jul 7;10(7):e70428. doi: 10.1002/hem3.70428 (PMC13340139; doi:10.1002/hem3.70428)
Supplement: Supplementary file 12 — Supporting Information. [file HEM3-10-e70428-s007.docx]

*Supplementary Table 10. Overall survival and cytokine values*

| variable_name | hazard_ratio | CI_lower | CI_upper | p_value | p_adj |
| --- | --- | --- | --- | --- | --- |
| CCL8 | 1.3 | 0.91 | 1.9 | 0.15 | 0.380 |
| IL33 | 1 | 0.76 | 1.3 | 0.98 | 0.980 |
| CXCL12 | 1.2 | 0.58 | 2.6 | 0.59 | 0.840 |
| OLR1 | 1.1 | 0.89 | 1.4 | 0.32 | 0.530 |
| IL27 | 1.2 | 0.89 | 1.5 | 0.27 | 0.490 |
| IL2 | 0.96 | 0.73 | 1.3 | 0.78 | 0.900 |
| CXCL9 | 1.1 | 0.79 | 1.4 | 0.71 | 0.880 |
| TGFA | 1 | 0.68 | 1.6 | 0.86 | 0.940 |
| IL1B | 1.3 | 1.1 | 1.6 | 0.014 | 0.110 |
| IL6 | 1.2 | 0.96 | 1.4 | 0.14 | 0.370 |
| IL4 | 0.79 | 0.59 | 1.1 | 0.12 | 0.370 |
| TNFSF12 | 0.56 | 0.26 | 1.2 | 0.13 | 0.370 |
| TSLP | 1.2 | 0.94 | 1.6 | 0.14 | 0.370 |
| CCL11 | 0.95 | 0.56 | 1.6 | 0.86 | 0.940 |
| HGF | 1.6 | 1.1 | 2.5 | 0.019 | 0.110 |
| FLT3LG | 0.9 | 0.72 | 1.1 | 0.31 | 0.530 |
| IL17F | 1.1 | 0.92 | 1.4 | 0.25 | 0.460 |
| IL7 | 0.96 | 0.77 | 1.2 | 0.74 | 0.880 |
| IL13 | 0.99 | 0.84 | 1.2 | 0.92 | 0.960 |
| **IL18** | **1.9** | **1.3** | **2.8** | **0.002** | **0.030** |
| CCL13 | 1.2 | 0.89 | 1.7 | 0.2 | 0.440 |
| TNFSF10 | 0.95 | 0.48 | 1.9 | 0.87 | 0.940 |
| CXCL10 | 1.1 | 0.8 | 1.4 | 0.66 | 0.850 |
| IFNG | 1.1 | 0.82 | 1.4 | 0.62 | 0.840 |
| **IL10** | **1.4** | **1.1** | **1.6** | **0.00087** | **0.020** |
| CCL19 | 1.2 | 0.87 | 1.7 | 0.24 | 0.460 |
| TNF | 1.4 | 0.86 | 2.4 | 0.17 | 0.390 |
| IL15 | 2.2 | 1.3 | 3.9 | 0.0056 | 0.063 |
| CCL3 | 1.4 | 1 | 1.8 | 0.023 | 0.110 |
| **CXCL8** | **1.4** | **1.1** | **1.7** | **0.00057** | **0.020** |
| MMP12 | 1.1 | 0.82 | 1.6 | 0.45 | 0.720 |
| CSF2 | 1.2 | 0.91 | 1.7 | 0.17 | 0.390 |
| CSF3 | 1.4 | 0.96 | 1.9 | 0.085 | 0.320 |
| VEGFA | 0.92 | 0.63 | 1.3 | 0.65 | 0.850 |
| IL17C | 1.1 | 0.83 | 1.4 | 0.54 | 0.810 |
| EGF | 0.95 | 0.83 | 1.1 | 0.47 | 0.730 |
| CCL2 | 1.4 | 1.0 | 2.0 | 0.025 | 0.110 |
| IL17A | 1.3 | 1.0 | 1.5 | 0.024 | 0.110 |
| OSM | 1.2 | 0.94 | 1.6 | 0.13 | 0.370 |
| CSF1 | 1.7 | 0.69 | 4.3 | 0.25 | 0.460 |
| CCL4 | 1.3 | 1.0 | 1.7 | 0.052 | 0.210 |
| CXCL11 | 1.1 | 0.85 | 1.3 | 0.61 | 0.840 |
| LTA | 1.1 | 0.57 | 2.2 | 0.74 | 0.880 |
| CCL7 | 1 | 0.79 | 1.3 | 0.97 | 0.980 |
| MMP1 | 0.79 | 0.66 | 0.96 | 0.017 | 0.110 |

Each of these analyses is a Cox regression on the individual log cytokine value, adjusted for age and sex.
